# Versatile Nanoplatforms with enhanced Photodynamic Therapy: Designs and Applications

**DOI:** 10.7150/thno.46288

**Published:** 2020-06-05

**Authors:** Kai Yan, Yabin Zhang, Chenglong Mu, Qunna Xu, Xunan Jing, Daquan Wang, Dongfeng Dang, Lingjie Meng, Jianzhong Ma

**Affiliations:** 1 College of Bioresources Chemical and Materials Engineering, Shaanxi University of Science and Technology, Xi'an 710021, China.; 2 School of Science, Xi'an Key Laboratory of Sustainable Energy Material Chemistry, MOE Key Laboratory for Nonequilibrium Synthesis and Modulation of Condensed Materials, Xi'an Jiaotong University, Xi'an 710049, P. R. China.; 3 Key Laboratory of Testing Technology for Manufacturing Process of Ministry of Education, Southwest University of Science and Technology, Mianyang 621010, P. R. China.; 4 Institute of Textiles & Clothing, The Hong Kong Polytechnic University, Hung Hom, Kowloon, Hong Kong, China.

**Keywords:** photodynamic therapy, malignant tumor, versatile nanoplatforms, microenvironment

## Abstract

As an emerging antitumor strategy, photodynamic therapy (PDT) has attracted intensive attention for the treatment of various malignant tumors owing to its noninvasive nature and high spatial selectivity in recent years. However, the therapeutic effect is unsatisfactory on some occasions due to the presence of some unfavorable factors including nonspecific accumulation of PS towards malignant tissues, the lack of endogenous oxygen in tumors, as well as the limited light penetration depth, further hampering practical application. To circumvent these limitations and improve real utilization efficiency, various enhanced strategies have been developed and explored during the past years. In this review, we give an overview of the state-of-the-art advances progress on versatile nanoplatforms for enhanced PDT considering the enhancement from targeting or responsive, chemical and physical effect. Specifically, these effects mainly include organelle-targeting function, tumor microenvironment responsive release photosensitizers (PS), self-sufficient O_2_ (affinity oxygen and generating oxygen), photocatalytic water splitting, X-rays light stimulate, surface plasmon resonance enhancement, and the improvement by resonance energy transfer. When utilizing these strategies to improve the therapeutic effect, the advantages and limitations are addressed. Finally, the challenges and prospective will be discussed and demonstrated for the future development of advanced PDT with enhanced efficacy.

## Introduction

Cancer is one of the greatest conundrums in the modern medical field, posing a great threat to human health 1-3. Conventional cancer therapies include surgery 4, chemotherapy 5, and radiotherapy 6, which have pros and cons. Surgery fails to completely remove all tumor tissues, resulting in a high recurrence rate 7. Chemotherapy usually lacks selectivity to cancer cells and typically causes multi-drug resistance 8. Radiotherapy can damage healthy cells and tissues near cancer cells 9. Thus, existing cancer treatment options have limited therapeutic effects. Recently, new treatment modalities with improved efficacy and reduced adverse effects have received much attention, especially phototherapy dating back to three thousand years. The treatment of lupus vulgaris with a short-wavelength light by Niels Ryberg Finsen was awarded the Nobel Prize in 1903, instigating the development of phototherapy 10, which is a minimally invasive, controllable technique that relies on the phototherapeutic agents in conjunction with light irradiation to kill cancer cells selectively 11-13. As two typical phototherapy approaches, photodynamic therapy (PDT) and photothermal therapy (PTT) have been extensively studied and are commonly used. PTT agents absorb a selective wavelength of light and dissipate the absorbed energy through non-radiative decay that increases the temperature in the local environment, leading to the thermal ablation of cancer cells 14, 15. However, in the actual treatment process, excessive heat tends to damage normal cells and tissues, causing complications and negatively affecting the treatment.

Despite its shortcomings, PDT has become a promising therapeutic option by using photosensitizers (PS) and light irradiation to induce cell death and tissue destruction 16. Upon exposure to appropriate wavelength light, the PS can transfer energy to surrounding oxygen molecules and produce cytotoxic reactive oxygen species (ROS), such as singlet oxygen (^1^O_2_), superoxide, hydrogen peroxide (H_2_O_2_), and hydroxyl radicals, which efficiently eliminate highly proliferating cells by severe and irreversible damage to their organelle 17, 18. PDT has been extensively studied in the treatment of both premalignant and malignant skin tumors 19, 20, head 21 and neck 22, 23, prostate 24, 25, non-small-cell lung 26, 27, and other cancers. Due to the high cytotoxicity of the generated ^1^O_2_, PDT can generate much greater treatment effects than PTT. Compared to the widely used chemotherapy and radiotherapy, PDT entails beneficial features like reduced side effects on normal tissues, possible repeatable doses, and absence of scarring 28, 29. Considering these merits, PDT has been under the research spotlight for in-depth investigation as an important paradigm for a variety of cancers and non-cancerous lesions in recent years.

Despite considerable progress in its use for cancer treatment, a few outstanding issues for the efficacy of PDT include PS utilization efficiency, light penetration depth, and endogenous oxygen generation in tumors 30-32. The hypoxia in tumor tissues, caused by the abnormal vasculature perfusion, uncontrollable cell proliferation, and dysfunctional lymphatic system, significantly impairs the PDT outcome 33, 34. Conversely, PDT aggravates the hypoxia in the tumor microenvironment because of oxygen depletion and the vascular shutdown effect, finally triggering tumor metastasis and tumor recurrence 35, 36. Besides, the antioxidative glutathione (GSH) overexpressed in cancer cells can scavenge the generated ROS, thus lowering PDT efficacy. To address these issues and deliver the PS molecules to the desired site, several new approaches have been considered for maximizing the efficacy of PDT. The integration of nanotechnology with material science has led to the extensive application of nanoplatforms in medicine 37-39, especially nanoplatform-based drug delivery systems. These systems show diagnostic and therapeutic prospects in clinical applications due to the localization ability, high-efficiency, low-toxicity, and controlled release of drugs 40, 41. More specifically, PDT, combined with nanosystems has become a hot spot in the prevention, diagnosis, treatment, and monitoring of tumors. Such nanoplatforms are elaborately constructed to achieve enhanced PDT for specific targeting, high drug loading, and multi-functional integration 42-44.

**Figure 1:**
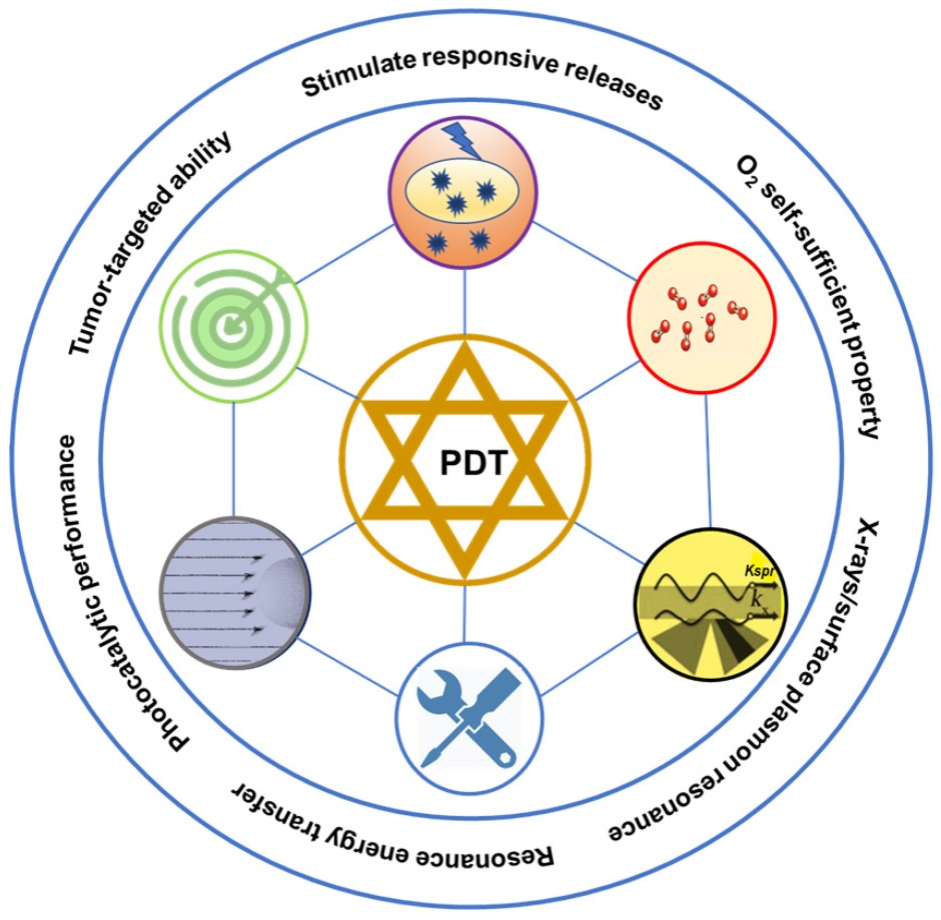
Schematic illustration of the versatile nanoplatforms for enhanced PDT.

This review aims to provide an overview of nanotherapeutic advancements for enhanced PDT targeted towards tumor therapy in the last five years (Figure 1). We categorize functional polymer nanoplatforms to achieve specific accumulation of PS in tumors to enhance PDT, such as targeting performance, responsive release, and charge turnover. High-efficiency PDT of self-oxygenation systems (carrying oxygen and generating oxygen) and photocatalytic hybrid nanoplatforms are summarized. Also, enhanced PDT with nanoplatforms using X-rays, plasmon resonance and other physical methods have been outlined. Finally, we highlight the challenges and possible developments of nanoplatforms for enhanced PDT, which could inspire the design of nanoplatforms and prompt clinical applications of PDT in tumor therapy.

## Targeted and responsive nanoplatforms for enhanced PDT

### Targeting nanoplatforms

When the nanoplatforms are injected into the biological tissue, they are often recognized as intruders by the immune system, provoking an immune response 45. Also, the nanoplatforms are likely to be picked up and cleared out by the mononuclear phagocyte system during blood circulation, leading to shortened circulation time and limited therapeutic effects 46. The nontargeting nanoplatforms can passively target the tumor through enhanced permeability and retention (EPR) effect and, therefore, can deliver a small amount of nanomedicine to the tumor sites. On the other hand, nanoplatforms endowed with targeting ability can achieve specific and improved delivery to tumor tissues. Another important aspect of PDT is that the PS is taken up by different subcellular organelles such as the mitochondria, lysosomes, endoplasmic reticulum, Golgi apparatus, and plasma membranes 47, 48 and under light irradiation, its subcellular localization largely determines the damage produced by ROS. Thus, it would be of great interest to exploit targeted nanoplatform delivery systems to transport PS directly into the desired subcellular organelles 49.

**Figure 2:**
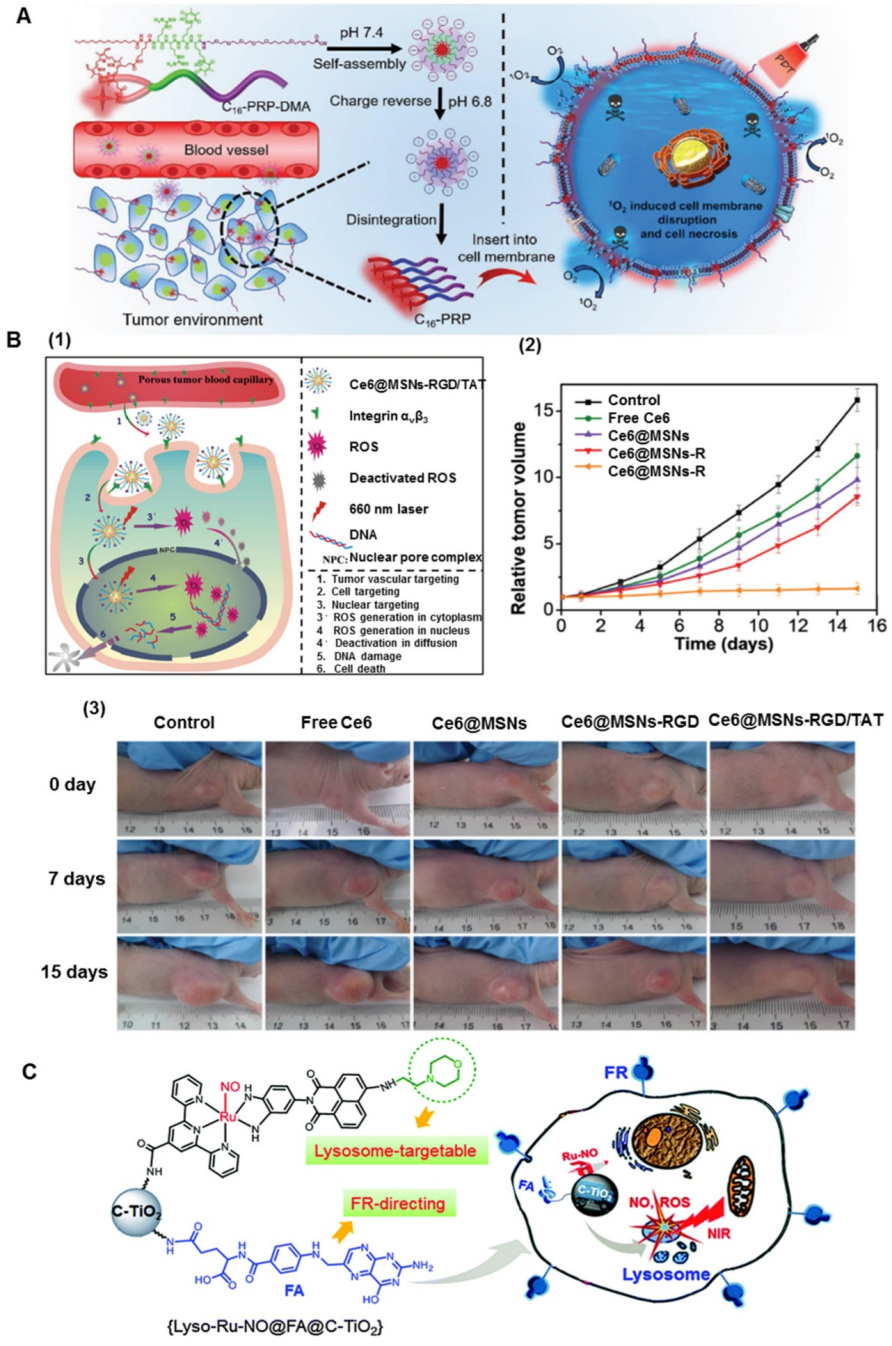
** A.** Membrane-anchoring PS for enhanced PDT. Self-assembly of chimeric peptides into nanoparticles and the charge reverse at tumor environment (left), resulting in cell membrane disruption and cell necrosis (right). Adapted with permission from ref. 53. Copyright 2017 Wiley-VCH. **B.** Intranuclear PS delivery and PS for enhanced PDT. (1) Schematic illustration of sequential-targeted PDT based on Ce6@MSNs-RGD/TAT. (2) Tumor growth curves of different groups of tumor-bearing mice after PDT. (3) Photographs of the mice taken after 0 day, 7 and 15 days of PDT. Adapted with permission from ref. 56. Copyright 2014 Wiley-VCH. **C.** Schematic representation of nanoplatforms and the directed attack of cancer cell lysosomes by NO and ROS under near infrared (NIR) light irradiation. Adapted with permission from ref. 66. Copyright 2016 Royal Society of Chemistry.

**Cell membrane** is the most important protective barrier in living cells maintaining cell integrity and guarantees the essential cellular functions 50, 51. Severe damage to the plasma membrane induces cellular apoptosis, necrosis, and autophagy. Cell membrane-targeted therapy can be more efficient because it does not require endocytosis of drugs. Moreover, many shortcomings of conventional chemotherapy, such as the intracellular degradation of drugs or drug resistance-induced failures, can be avoided 52. A novel charge reversible self-delivery chimeric peptide C_16_-PRP-DMA (C_16_-K(PpIX)RRK(DMA)K(DMA)-PEG-COOH) was designed for long-term cell membrane**-**targeted PDT (Figure 2A). The self-assembled nanoparticles could stay on the cancer cell membrane for a long time because of the synergetic effect of alkyl chain palmitic acid and positively charged RRKK attached to the cell membrane 53. Under light irradiation, the ROS generated by the inserted C_16_-PRP-DMA directly disrupted the cell membrane and rapidly caused cell necrosis, remarkably increasing the PDT effect *in vitro* and *in vivo*. To maximize the therapeutic potency, a self-transformable pH-driven, membrane-anchoring nanoplatform was constructed by attaching protoporphyrin IX (PpIX) to the end of the water-soluble peptide 54. Benefiting from the insertion peptide, the nanoplatform could form an α-helix structure under acidic conditions (pH 6.5 or 5.5), but remained random coil at normal pH of 7.4. This feature enabled successful insertion of nanoplatforms into the membrane lipid bilayer, especially for cancerous cell membranes in the acidic tumor microenvironment. Under laser irradiation at 630 nm, the plasma membrane was severely damaged by in situ generated ROS, subsequently inducing cell death 54. In particular, *in vivo* studies indicated the high inhibition effect of this nanoplatform against primary tumors and the successful prevention of tumor metastasis.

**Nucleus** is the most significant organelle in the cell and contains most of the intracellular genetic materials 55. Any disruptions inside the nucleus would subsequently affect the cellular DNA disturbing the highly regulated cell cycle. Consequently, nuclear-targeted strategies could facilitate the penetration of the PS into the nucleus and greatly improve therapeutic efficiency. As shown in Figure 2B, Shi's group *et al.* made TAT and RGD peptides co-conjugated with mesoporous silica nanoparticles (MSNs) as PDT carriers 56. TAT peptides enabled the nuclear penetration of mesoporous silica nanoparticles for efficient accumulation of the PS inside the nuclei. Upon irradiation, the intranuclearly accumulated PS could generate ROS to destroy DNA instantaneously. Moreover, the co-conjugated RGD peptides endowed the nuclear-targeted delivery system with recognition of and specific binding to tumor vasculature and tumor cell membranes for significantly enhanced specificity and reduced side effects. As is evident from Figures 2B2 and 2B3, the nuclear-targeted nanoplatforms exerted substantially stronger inhibition effect on tumor growth than Ce6@MSNs-RGD, Ce6@MSNs, and free Ce6. Taking advantage of structural programmability and biocompatibility, self-assembled DNA nanostructures have attracted much attention in biomedical applications. Yang *et al.* developed nanoscale coordination polymers (NCPs) based on the interaction between calcium ions (Ca^2+^) and AS1411 DNA G quadruplexes 57. Both Ce6 and an iron-containing porphyrin inserted into the G-quadruplex structure. The NCPs enabled the intranuclear transport of Ce6 to generate ROS inside cell nuclei. AS1411 inhibited the expression of antiapoptotic protein B-cell lymphoma 2 (Bcl-2), allowing for greatly improved PDT-induced cell apoptosis. Furthermore, the catalase-mimicking DNA enzyme function of G-quadruplexes and hemin could react with tumor endogenous H_2_O_2_ to generate oxygen for enhanced PDT by overcoming the hypoxia-associated resistance.

**Mitochondria** is the primary source of cellular ROS production (approximately up to 90%), which plays vital roles in energy production and cell apoptosis 58, 59. Moreover, mitochondria could be susceptible to excessive ROS. The initial formation of ROS-induced changes in mitochondrial permeability, depolarization, deformation, and release of cytochrome C, ultimately resulted in apoptosis 60, 61. Therefore, a PS delivery system targeting mitochondria is greatly beneficial for enhancing the photodynamic therapeutic effect. The peptide Ad-CGKRK-GFLG-EE-HAIYPRH(T7) with a guest molecule Ad termination and cathepsin B (CTSB) (overexpressed in cancer cells)-cleavable linker GFLG was developed, and Ce6 was conjugated to the host molecule b-Cyclodextrin (b-CD) through an amide bond. Through CD/Ad recognition, CD-Ce6 and Ad-CGKRK-GFLG-EE-HAIYPRH (T7) inclusion complexation self-assembled into supramolecular nanoplatforms 62. T7 modification endowed supramolecular nanoplatforms with targeting ability for MCF-7 cancer cells. After internalization, the cleavage of the GFLG sequence by overexpressed CTSB exposed the mitochondria-targeting peptide CGKRK allowing the accumulation of nanocarriers in mitochondria and enhancing the PDT effect. Another mitochondria and plasma membrane dual-targeting nanoplatform was developed using the chimeric peptide for single-agent synergistic PDT 63, which included a hydrophobic photosensitizer PpIX, a bioactive peptide sequence with dual targeting function, and a hydrophilic poly (ethylene glycol) (PEG) chain. This amphipathic chimeric peptide could form spherical micelles by self-assembly in aqueous solution, with high drug loading efficacy as well as the excellent ability to produce ROS. Plasma membrane-targeting PDT increased the membrane permeability to improve the cellular delivery of nanoplatforms, and even directly disrupted the cell membrane to induce cell necrosis. Additionally, mitochondria-targeted PDT reduced mitochondrial membrane potential and significantly promoted cell apoptosis. Consequently, the dual-targeting property facilitated the effective subcellular localization of PpIX to generate ROS for enhanced PDT.

**Lysosomes** are important subcellular acidic organelles for cellular homeostasis, whose dysfunction is responsible for several diseases 64. There is ample evidence to verify that the lysosomal death pathway could contribute to the programmed cell death sensitivity of cancer cells 65. Xiang *et al.* developed novel cancer cell lysosome-targetable NO-delivery nanoplatforms composed of a ruthenium nitrosyl donor (Lyso-Ru-NO), a cancer cell directing group of folic acid, and a carrier of biocompatible carbon-doped titanium dioxide nanoparticles (Figure 2C) 66. The ruthenium nitrosyl Lyso-Ru-NO group contained the Lyso-NINO ligand, wherein a morpholine moiety targeted the lysosomes. The incorporation of folic acid and morpholine groups rendered it capable of targeting folate-receptor overexpressing-cancer cells and specifically accumulating in the subcellular lysosomal organelles, where 808 nm NIR light irradiation simultaneously released NO and ROS. The cytotoxicity assay showed that the dual-targeted nanoplatforms have the highest anticancer efficacy compared to their nontargeted counterparts under NIR light sensitization. To further compare differences in mitochondria- and lysosome-targeted PDT, two PS were designed based on iridium (III) complex (Ir-P(ph)_3_ and Ir-alkyl group, which specifically targeted the mitochondria and lysosomes, respectively. The results indicated that HeLa cells treated with the mitochondria-targeted complex kept a slower respiration rate, resulting in a higher intracellular oxygen level under hypoxia. Consequently, this complex showed an enhanced PDT effect compared to the lysosome-targeted complex, especially under hypoxic conditions 67. It is possible that the mitochondria-targeted PS inhibited mitochondrial respiration, leading to higher intramitochondrial oxygen content, and, therefore, is more beneficial for PDT in hypoxic tumor cells.

Apart from organelle-targeting nanoplatforms, other nanoplatforms can also achieve enhanced PDT. Macrophages are highly abundant in the stroma of solid tumors and have been demonstrated to play an important role in the development and progression of cancer 68. Moreover, macrophages can enrich much more PS than tumor cells, making the PDT effect greatly potentiated when the tumors are treated with PS with a macrophage-activating factor. C-Phycocyanin, a macrophage-targeting agent, has been widely used in food, cosmetics, and biomedical applications due to its good biocompatibility, non-toxicity, water-solubility, unique color, strong absorption in the visible region (550-650 nm), and high fluorescence quantum yield. When combined with unsubstituted zinc phthalocyanine, it could facilitate PS for PDT. Furthermore, this conjugation exhibited an enhanced photodynamic effect due to the improved solubility and aggregation of zinc phthalocyanine 69. Thus, C-Phycocyanin can act as both a new class of tumor-associated macrophages PS and a desirable nanoplatform for other therapeutic agents. Besides, lactoferrin is a member of the transferrin family and commonly found in blood plasma, and has a potential role in treating diseases because of its antioxidant, antitumor, antiviral, and antifungal properties. Adimoolam *et al.* loaded Ce6 into lactoferrin by the water-in-oil emulsion method 70. The yield of reactive oxygen was enhanced in the nanoplatforms compared to free Ce6. Also, specific Ce6 release at low pH and higher uptake and intracellular concentrations of Ce6 compared with free Ce6* in vitro* were observed. Upon exposure to light, the nanoplatforms caused light-mediated cell death in the SK-OV-3 and MDA-MD 231 cells. Compared to free Ce6, it showed a substantial decrease (44 times) in the Ce6 requirement.

Despite significant advancements, targeted therapeutic agents are still limited to clinical applications. The development of malignant tumors is an extremely complicated process. It involves multiple genes and new gene mutations evolve in a multistep process, increasing the difficulty of targeted therapy. Targeted tumor agents show considerable toxicity to the digestive tract and vasculature with a negative impact on the therapeutic effect. To overcome the defects in targeted therapy, the joint application of various targeted drugs might show a better outcome.

### Responsive nanoplatforms for enhanced PDT

Efficient cellular internalization of the nanoplatforms and on-demand release of the PS are two significant steps for efficacy enhancement 71, 72. A high degree of cellular uptake can guarantee the maximal amount of intracellular PS, whereas rapid cargo release can address the problem from the limited diffusion distance as well as the short half-life of ^1^O_2_. It is vital to design responsive nanoplatforms to enhance cellular uptake and control PS release. So far, exogenous (externally applied) stimuli to trigger PS release, such as temperature 73, 74, light 75, 76, and magnetic triggers 77, 78, have been explored widely. Besides, tumor pathophysiology displays characteristic changes such as pH 79, enzyme activity 80, or redox properties 81, and allowing opportunities to exploit these endogenous factors as internal stimuli. Table 1 summarizes targeted and responsive nanoplatforms for enhanced PDT.

#### Tumor microenvironment (pH and GSH)-responsive nanoplatforms

**Figure 3:**
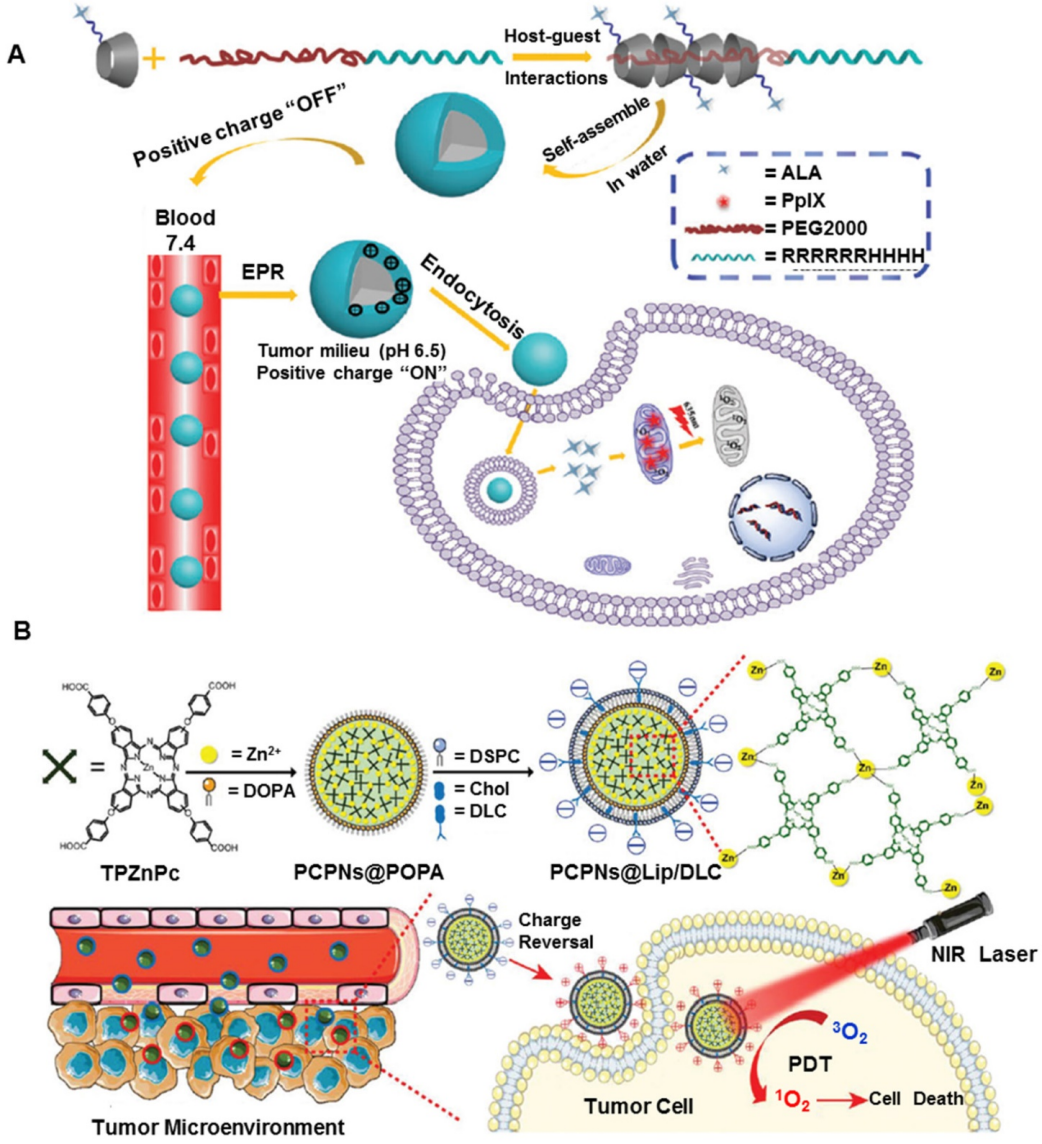
** A.** Schematic illustrations of ALA pseudopolyrotaxane prodrug micelles. Adapted with permission from ref. 88. Copyright 2016 Royal Society of Chemistry. **B.** Schematic illustrations of PCPNs@Lip/DLC for enhanced PDT. Adapted with permission from ref. 93. Copyright 2017 Royal Society of Chemistry.

The microenvironment of cancerous tissues is specific and markedly different from that of the extracellular matrix and normal tissues. pH is one of the stimuli that has been most frequently utilized to control intracellular drug delivery in specific organs based on environmental changes induced by pathological conditions 82, 83. Furthermore, the pH difference can be found in some organelles, such as endosomes (pH lower than 4.5) and lysosomes (~pH 5.5), where it is much lower than other intracellular organelles 84, 85. Due to the pH gradient between the tumor microenvironment (pH ≈ 6.5) and the physiological environment (pH ≈ 7.4), the pH-responsive nanoplatforms undergo conformational changes through various mechanisms, such as protonation 86, charge reversal, or cleavage of a chemical bond 87, facilitating tumor-specific cell uptake or drug release. Such responsive nanoplatforms are usually fabricated through physically encapsulating or chemically conjugating the PS. So far, a variety of pH-responsive nanoplatforms have been designed to exploit low extracellular pH_ex_ or endosomal pH_en_. For example, Tong *et al* attached 5-aminolevulinic acid (ALA) to a-cyclodextrin (a-CD) through an acid-labile hydrazone bond. Subsequently, the pH-responsive, cell-penetrating peptide R6H4 (RRRRRRHHHH) was conjugated to PEG to form PEG-R6H4. As shown in Figure 3A, dual pH-responsive ALA pseudopolyrotaxane prodrug micelles were developed by the host-guest interaction of a-CD and PEG. Taking advantage of the pH-responsive R6H4, the nanoplatforms were easily internalized into tumor cells. Furthermore, ALA was released by the cleavage of the hydrazone bond at endo/lysosomal pH and further converted to PpIX for enhanced PDT 88. To achieve multifunctionality, Chu *et al* used pH-responsive copolymers poly(ethylene glycol) methacrylateco-2-(diisopropylamino)ethyl methacrylate, biodegradable copolymers methoxypoly(ethyleneglycol)/poly(ε-caprolactone), and maleimide-modified biodegradable copolymers to prepare new micelles. After entrapping the photosensitizer Ce6, the micelles were coated with the epidermal growth factor receptor (EGFR)-targeting peptide GE11, and showed enhanced PDT efficacy 89. This could be attributed to the increased Ce6 uptake due to GE11 targeting and increased release of Ce6 for improved elimination of cancer cells in the acidic tumor microenvironment.

Recently, pH-dependent charge reversal delivery nanoplatforms exhibited great superiority in controlled release and targeted drug delivery 90. The surface charge of nanoplatforms plays a decisive role in cell internalization and blood stability. The nanoplatforms can keep their original negatively charged status under neutral conditions in the bloodstream, thus preventing nonspecific interactions with serum proteins and normal tissues 91. Upon arriving at the tumor tissues or endo/lysosomes, the original negative charge can quickly convert into positively charged status, thus triggering the efficient cell internalization. For example, tumor pH-sensitive photodynamic nanoplatforms NaYF_4_:Yb,Er@CaF_2,_ were comprised of self-assembled, PS-grafted, pH-responsive polymeric ligands 92, which were prepared by derivatizing poly(ethylene glycol)-poly(β-benzyl-l-aspartate) with 1-(3-aminopropyl) imidazole, 3-phenyl-1-propylamine, and Ce6. The nanoplatforms were negatively charged without any discernible photoactivity at normal blood pH of ≈7.4 but quickly switched their surface charge from negative to positive at an extracellular tumor pH of ≈6.5. These nanoparticles not only exhibited enhanced tumor-cell internalization due to charge reversal but disassembled into well-dispersed nanoparticles in the endo/lysosomes of tumor cells, thus enabling efficient PDT. Besides, charge-reversal phthalocyanine-based coordination polymer nanoplatforms (PCPNs@Lip/DLC) were developed to improve the curative effect of PDT 93. Tetra(4-carboxyphenoxy)-phthalocyaninatozinc coordinated with zinc was coated with a self-assembled lipid bilayer (Figure 3B), and 1,2-dicarboxylic-cyclohexane anhydride-modified lysyl-cholesterol (DLC) was functionalized on the surface of PCPN, endowing it with a charge-reversal ability. When exposed to a mildly acidic environment in the tumor tissue, DLC could degrade and enabled increased tumor uptake of PCPN due to the electrostatic interaction with the negatively charged cell membrane. The nanoplatforms were verified to enhance tumor cellular uptake and generate increased intracellular ROS after irradiation. As confirmed by *in vitro* and *in vivo* studies, the nanoplatforms remarkably increased the PDT effect. Despite the obvious potential of pH-sensitive nanoplatforms, they sometimes need to be combined with other stimuli to achieve precise and specific drug release at the targeted tumor sites.

**Figure 4:**
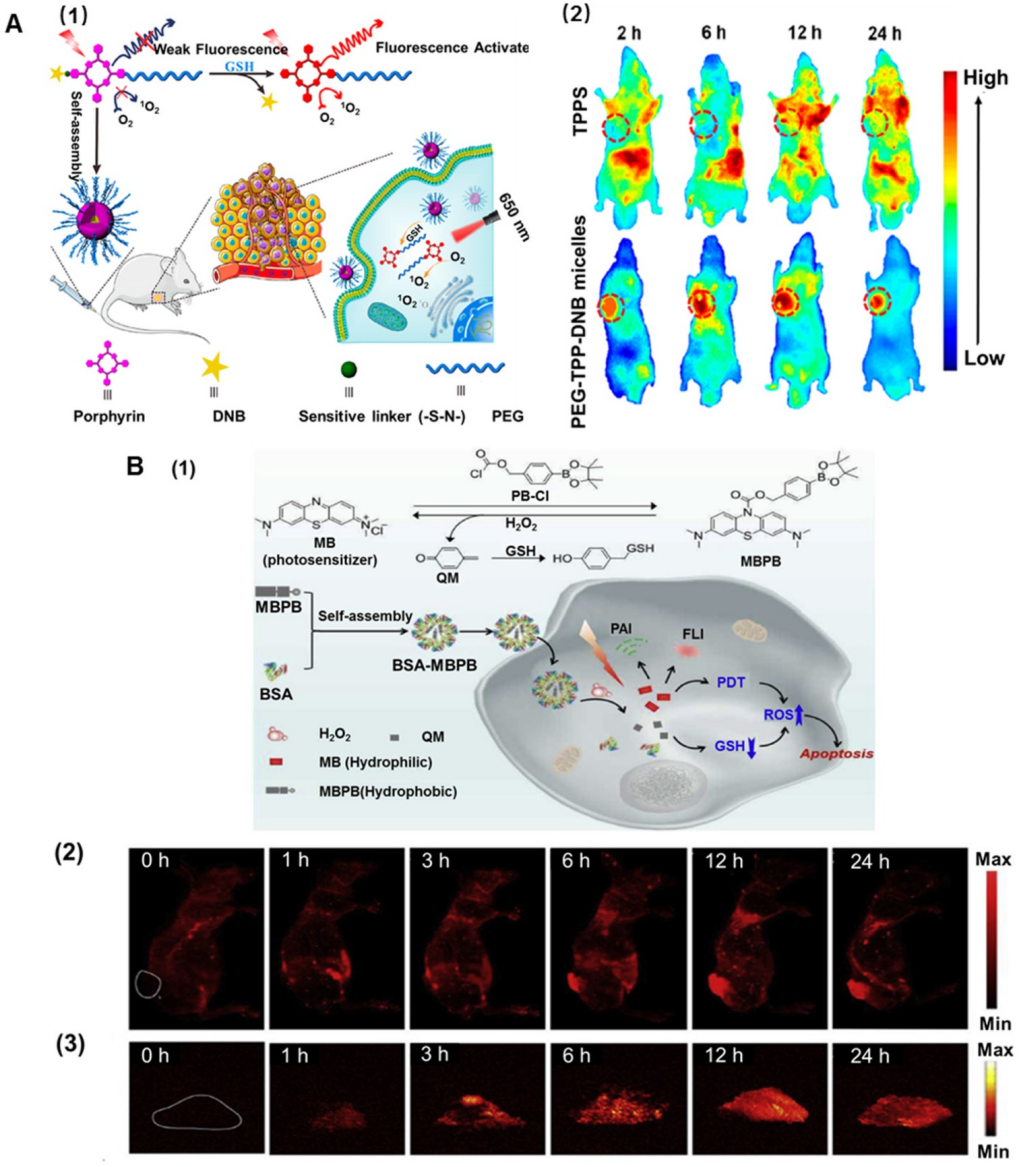
** A.** Redox amphiphile for enhanced PDT. Adapted with permission from ref. 102. Copyright 2019 American Chemical Society. (1) Schematic illustration of GSH-responsive intracellular activation of amphiphilic nanoplatforms and the tumor-targeted PDT*.* (2) *In vivo* fluorescence imaging of the 4T1 tumor-bearing mice after intravenous injection of 5,10,15,20-tetrakis (4-sulfophenyl) porphyrin (TPPS) and PEG-TPP-DNB micelles. **B.** H_2_O_2_-responsive biodegradable nanomedicine for imaging-guided PDT. Adapted with permission from ref. 106. Copyright 2019 Elsevier. (1) Schematic illustration of the synthesized photosensitizer (MBPB) and BSA-MBPB nanoplatforms, *in vivo* fluorescence (2) and photoacoustic imaging (3) before and after 1, 3, 6, 12 and 24 h post injection of nanoplatforms.

Besides the difference in pH environment, the intracellular glutathione (GSH) level of cancer cells has been widely reported to be considerably higher (about 10 mM) than that in the extracellular matrix (about 2 μM) 94, 95. GSH can be used as a reducing agent due to the thiol groups and is an important antioxidant that can prevent the damage to the cellular components induced by ROS 96. Previous reports have indicated that high intracellular GSH levels in tumor cells can effectively trigger drug release 97, 98, facilitating efficient PS uptake from the functional nanoplatforms. Disulfide, selenium, or tellurium-containing nanoplatforms were exploited as redox-responsive drug-delivery systems 99, 100. Zhang* et al.* developed supramolecular host-guest complexation between a PEG-functionalized pillararene and a pyridinium-terminated porphyrin derivative-bearing disulfide bond 5. The supramolecules could self-assemble into spherical micelles with good colloidal stability, which exhibited the rapid release of porphyrin PS in a reducing environment. *In vitro* cytotoxicity confirmed that the supramolecular amphiphiles exhibited superior cellular uptake and remarkably enhanced PDT 101. Another biocompatible stimuli-activated photosensitive nanoplatform (PEG-TPP-DNB) based on porphyrin molecules was designed (Figure 4A1). 5,15-bis(4-aminophenyl)-10,20-diphenylporphyrin was regarded as the center of molecular structure and further modified at both ends with PEG chain and the functional group 2,4-dinitrobenzene via pH-deionized and thiol-labile sulfonamide linkages. Because nitrobenzene has a strong ability to withdraw electrons on the porphyrin macrocycles, the nanoplatforms remarkably quenched the fluorescence and ^1^O_2_. Therefore, the redox-sensitive and activated nanoplatforms could minimize the biological imaging background and phototoxicity to normal tissues. In aqueous media, the amphiphilic nanoplatforms could self-assemble into nanomicelles and dissociate in response to the reductive thiol such as GSH. *In vivo* bioimaging further elucidated that the micelles showed selective tumor imaging and targeted PDT antitumor effect as well as low systemic toxicity (Figure 4A2). Overall, the amphiphilic nanoplatforms not only afforded targeted release of PS for maximized tumor enrichment but also exhibited excellent tumor-targeting PDT efficacy 102.

Although GSH stimulates the responsive nanoplatforms to release PS for enhanced PDT, the high concentration of GSH decreases ROS generation in cells, reducing the effectiveness of PDT. It has been demonstrated that a decrease in GSH concentration in tumor tissues could promote the ROS level by using nanoplatforms 103, 104. A nano-metal-organic framework (MOF) was designed to enhance PDT. Cu^II^ as the active center of MOF could specifically bind and absorb GSH, thus directly reducing the intracellular GSH concentration and increasing the ROS level. Simultaneously, the porphyrin ligand could be used as the PS to generate abundant ROS under light irradiation. *In vitro* experiments indicated that MOF was readily taken up by breast cancer cells, and high ROS levels were produced under light irradiation. This synergistically-increased ROS concentration accelerated apoptosis, enhancing the effect of PDT 105. Using a similar enhancement mechanism, Zeng *et al.* incorporated a p-phenylboronic ester (PB) into the clinical PS methylene blue with a carbonochloridate PB-Cl, and encapsulated them within BSA to achieve imaging-guided tumor-targeted effective PDT (Figure 4B1). In the presence of H_2_O_2_, the resultant caged nanoplatforms underwent H_2_O_2_-mediated boronic ester oxidation, releasing the hydrophilic methylene blue to produce ^1^O_2_, and quinoned methide rapidly deactivated GSH boosting the ^1^O_2_ yield, thus synergistically strengthening the efficacy of PDT. Furthermore, the BSA-MBPB nanoplatforms could also recover the fluorescent and photoacoustic dual-modal imaging of the tumor region and precisely guide the tumor-selective PDT 106 (Figure 4B2 and 4B3).

#### Enzyme-responsive nanoplatforms

Enzyme-responsive nanoplatforms have been increasingly utilized for PDT because they could selectively and efficiently involve in all physiological, metabolic, and pathological processes 107. Several studies have shown that hyaluronidase (HAase) enables the degradation of extracellular matrix to enhance therapies by increasing intratumor penetration of the drugs, and modulates the hypoxic tumor microenvironment 108. A smart hyaluronidase-activated theranostic nanoplatform was prepared based on hyaluronic acid (HA) coupled with Ce6 using adipic dihydrazide (ADH). This nanoplatform could be specifically degraded and released by hyaluronidase in the tumor, exhibiting higher fluorescence and photoacoustic intensity than free Ce6. Additionally, this HA-based nanoplatform showed more effective PDT than free Ce6 to suppress tumor growth *in vitro* and *in vivo* 109. Besides, the NQO1-based nanoplatforms also generated increasing attention as NQO1 is upregulated (~2-50-fold) in breast, pancreatic, colorectal, cervical, and lung cancers 110. Yao and co-workers developed self-assembled vesicles from amphiphilic block copolymers containing quinone trimethyl lock-capped self-immolative side linkages and quinone-bridged Nile blue in the hydrophobic block 111. Initially, their fluorescence emission and PDT potency were in the “off” state due to dye aggregation-caused quenching and quinone-rendered photoinduced electron transfer quenching. After internalization into NQO1-positive vesicles, the cytosolic NQO1 enzyme triggered the cleavage of quinone linkages and fluorogenic release of conjugated PS, resulting in NIR fluorescence emission turn-on and enhanced PDT.

#### ROS-responsive nanoplatforms

**Figure 5:**
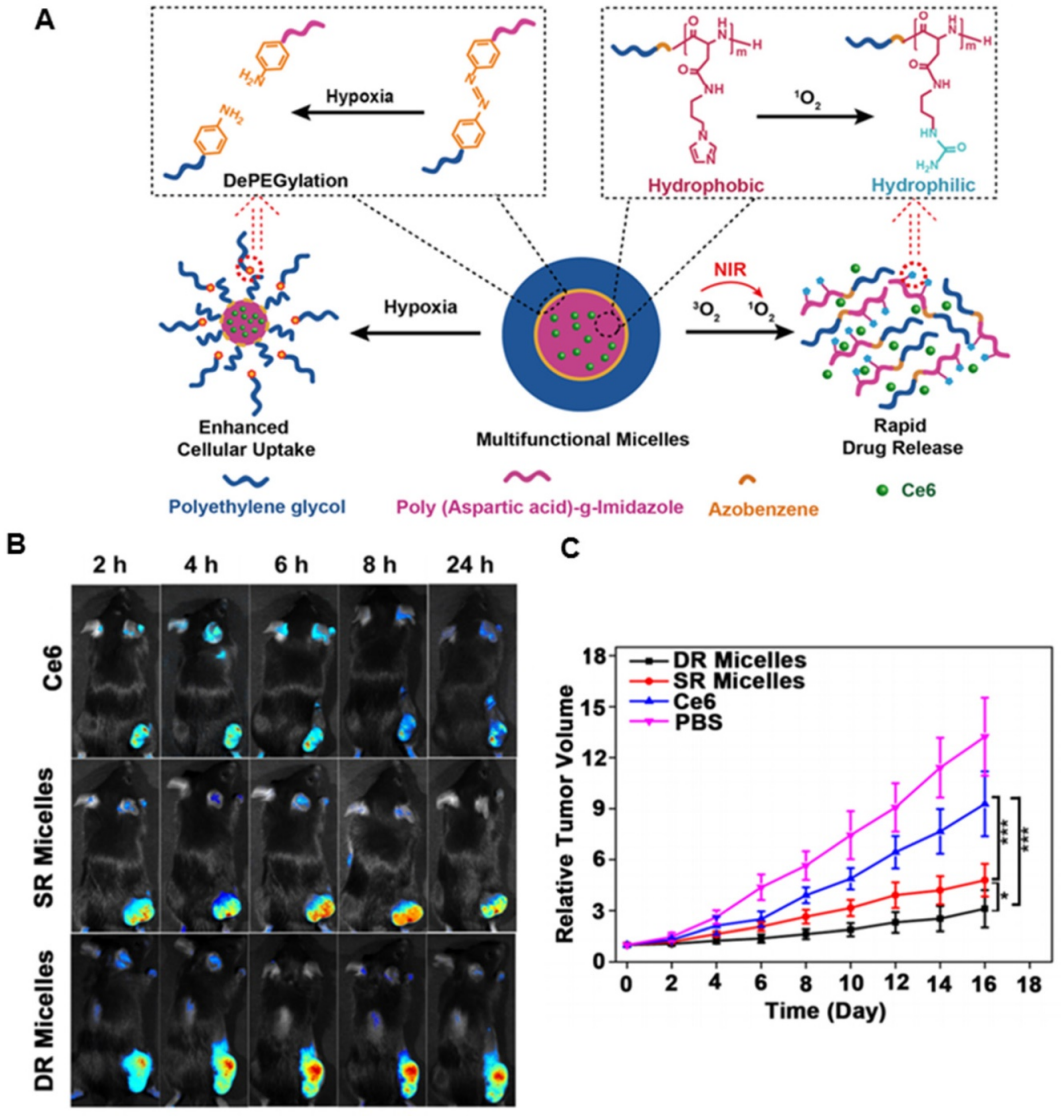
Polymeric micelles for enhanced PDT via interactively triggered PS delivery.** A.** Illustration of the interactively hypoxia- and singlet oxygen-sensitive tailor polymeric micelles. **B.** *In vivo* fluorescent imaging of the tumor and healthy organs up to 24 h after intravenous injection of free Ce6, Ce6-loaded SR micelles, and Ce6-loaded DR micelles. **C.** Relative tumor volume upon treatment with DR micelles and control formulations (SR micelles, free Ce6, and PBS). Adapted with permission from ref. 118. Copyright 2018 American Chemical Society.

ROS plays a vital role in the physiological and pathophysiological processes of the human body 112. It is also essential for the growth, development, and adaptation of organisms. In normal conditions, low concentrations of ROS regulate cell-signaling pathways and promote cell proliferation, whereas high levels of ROS induce oxidative stress that can disrupt the steady-state of cells and damage cellular components including membrane lipids, proteins, DNA, and other biological molecules 113, 114. Therefore, a lack of ROS or an excess of ROS may induce several abnormalities, including autoimmune, cardiovascular, and neurodegenerative diseases 115, 116. This abnormal biochemical alteration in the disease sites has inspired studies on unbalanced ROS levels for developing target-specific drug delivery systems. By utilizing ROS-responsive materials and linkers, various ROS-responsive drug delivery systems have been developed for therapeutic purposes. ^1^O_2_-responsive nanoplatforms are particularly appealing because of the highly reactive nature of ^1^O_2_ and the rapid onset of responsive cascade events. Guruswamy *et al.* designed a novel biocompatible visible-light-responsive amphiphilic poly(ethylene glycol)-block-poly(caprolactone)copolymer by incorporating an ^1^O_2_-sensitive vinyldithioether linker between the hydrophilic segments and the hydrophobic block 117. The amphiphilic micelles could disassemble the nanostructure due to ^1^O_2_ generated by visible light, and further release Ce6 and anticancer drug doxorubicin (DOX) for enhanced PDT. To further regulate the release of PS for improved PDT, Li *et al.* developed a methoxy poly(ethylene glycol)-azobenzene-poly(aspartic acid) copolymer conjugated with imidazole as the side chains 118. The azobenzene and imidazole are hypoxia- and singlet oxygen-responsive (SR) moieties, respectively. The facilitated cellular uptake of micelles was realized by triggering azobenzene collapse that provoked PEG shedding, while Ce6 could be released rapidly by micelle disassembly from imidazole oxidation (Figure 5A). The ^1^O_2_-mediated cargo release not only overcame the limited diffusion range and short half-life of ^1^O_2_ but also decreased the oxygen level, which could, in turn, enhance internalization and increase the intracellular Ce6 concentration. *In vivo* imaging study demonstrated that more Ce6 were accumulated in the tumor from dually responsive (DR) micelles in contrast to free Ce6 (Figure 5B). *In vivo* efficacy study indicated that the multifunctional micelles could maximize the PDT antitumor efficacy via interactively triggered delivery of the PS (Figure 5C).

#### Photothermal-responsive nanoplatforms

A photothermal responsive system induced by NIR light has gained acceptance for drug delivery in consideration of high efficiency, the noninvasive modality to tumor treatment, as well as the high spatial resolution of NIR light 119, 120. Photothermal-response nanoplatforms also contribute to diffusion therapy of PS. Importantly, this system exhibits synergistic cancer therapy associated with hyperthermia and heat-induced local drug release. Recent photothermal nanoplatforms mainly include noble metallic nanoparticles (gold nanocages 121, gold nanorods 122), semiconductor nanocrystals (CuS 123, WS_2_ 124, and MoS_2_ 125) as well as carbon-based nanoparticles (carbon nanotubes 126, graphene 127, and carbon nanospheres 128), organic dye molecules (ICG 129, IR780 130), and organic semiconducting polymer nanoparticles (polypyrrole 131, PEDOT:PSS 132). Fu *et al.* developed a novel tumor-targeting photothermal heat-responsive nanoplatform by using dopamine-reduced graphene oxide nanosheets (rGO-PDA), mesoporous silica-coated on the rGO-PDA surface to load Ce6, and hyaluronic acid as a gate-keeper as well as a targeting moiety 133. The nanoplatforms showed excellent photothermal conversion ability under near-infrared radiation and controllable Ce6 release with an NIR irradiation response. It could specifically deliver Ce6 to CD-44 over-expressing cancer cells for PDT. The combination of excellent NIR photothermal conversion ability and controllable Ce6 release conferred on the nanoplatforms enhanced photodynamic therapeutic efficiency.

## Chemical effects of nanoplatforms for enhanced PDT

The majority of the chemical factors that enhance the photodynamic effect are focused on overcoming the problem of hypoxia in tumor sites. Generally, nanoplatforms could load oxygen, or react with specific substances to produce oxygen in the tumor microenvironment, or produce oxygen under light stimulation.

### Self-supplying oxygen nanoplatforms

In recent years, many studies have reported that various nanoplatforms, such as perfluorocarbons 134, hemoglobin 135, catalase 136, and manganese dioxide derivatives 137, 138, delivered oxygen to tumor sites to overcome anoxic environments. These substances show an improved PDT effect, and their mechanism of operation is slightly different, as explained in detail below.

#### Oxygen affinity nanoplatforms

**Figure 6:**
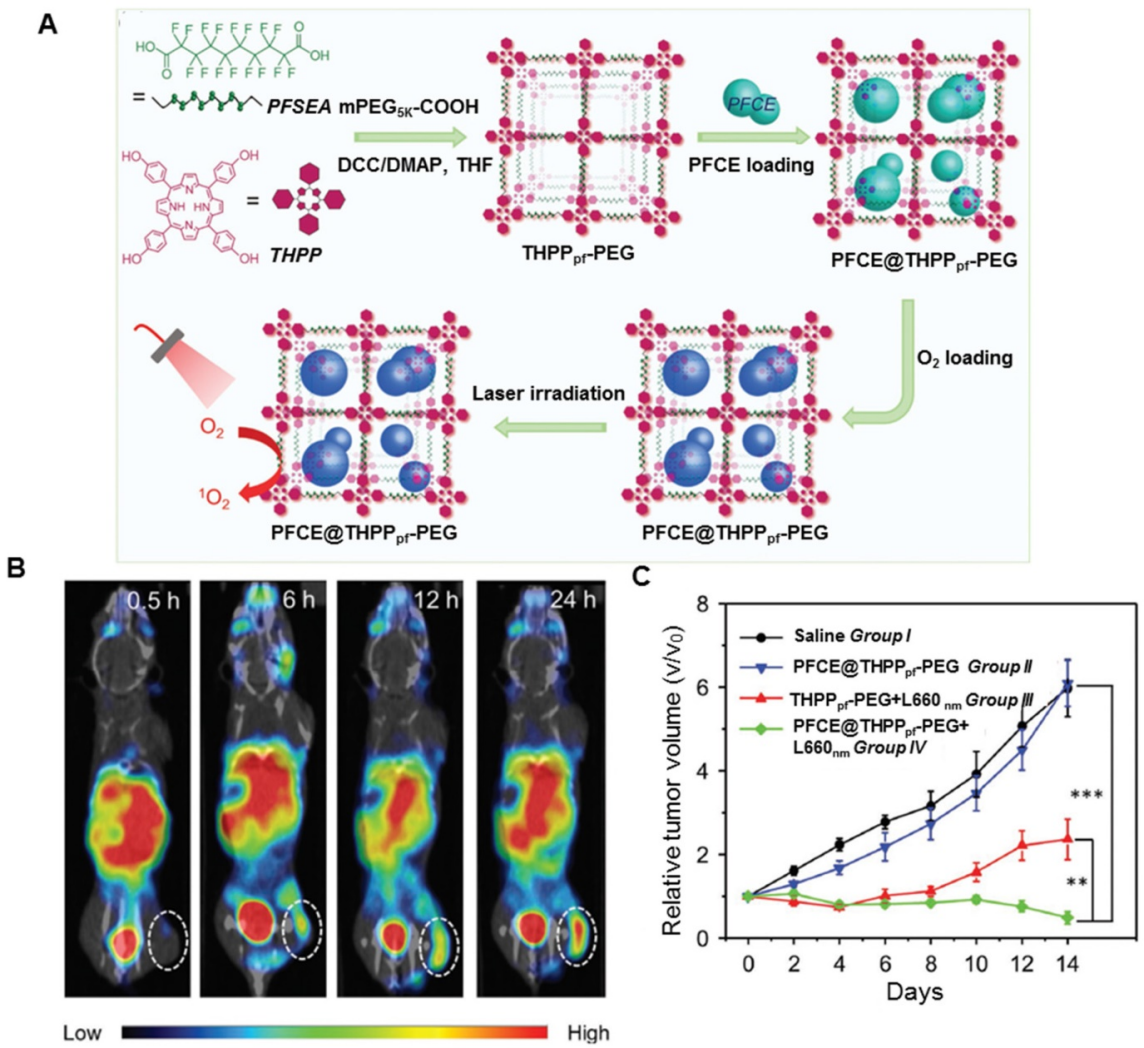
Covalent organic polymers for enhanced PDT. **A.** Scheme illustrating the synthesis route of THPP_pf_-PEG and the subsequent PFCE loading. **B.** SPECT images of 4T1 tumor-bearing mice with the injection of PFCE@THPP_pf_ (^99m^Tc)-PEG recorded at different time intervals. **C.** Tumor growth curves of four groups of mice after treatments as indicated. Adapted with permission from ref. 148. Copyright 2018 Wiley-VCH.

**Perfluorocarbons** are synthetic organic molecules, typically alkanes and their derivatives, whose hydrogen atoms are replaced by fluorine atoms 139, 140. They show biologic inertness and low toxicity even at high doses and, therefore, can be used for drug delivery. Because of the exceedingly favorable nuclear magnetic resonance properties and the virtual absence of fluorine in the human body, the perfluorocarbon carriers can also be utilized for ^19^F magnetic resonance imaging (MRI) 141. Furthermore, the unique electronic nature of C-F bonds endows the perfluorocarbon with excellent oxygen affinity, causing their oxygen-dissolving capacity to be much higher than that of other hydrocarbons or water and, therefore, have been widely explored for lung injury, emergency transfusion, and traumatic brain injury 142, 143. The ^1^O_2_ lifetime in perfluorocarbon (on the milli-second scale) is much longer than that in the cellular environment or in water (on the microsecond scale). Because perfluorocarbons have high oxygen solubility and ^1^O_2_ retention ability, they can maintain higher oxygen content than non-perfluorocarbon systems for potential oxygen self-enriched PDT 144-147. As displayed in Figure 6A, Liu's group designed fluorinated covalent organic polymers by cross-linking the photosensitizer meso-5, 10, 15, 20-tetra (4-hydroxylphenyl) porphyrin with perfluorosebacic acid and PEG by a one-pot esterification 148. The fluorinated nanoplatforms showed efficient loading of perfluoro-15-crown-5-ether (PFCE) due to the fluorinated chains of perfluorosebacic acid. After chelating with ^99m^Tc^4+^ radio-isotope utilizing the porphyrin structure of THPP, the fluorinated nanoplatforms could be tracked by *in vivo* SPECT imaging (Figure 6B). Finally, greatly enhanced photodynamic treatment effect was observed in mice after injecting PFCE@THPP_pf_-PEG because of the oxygen delivery by PFCE (Figure 6C). To increase the cell affinity and response of the fluorinated nanoplatforms, Ma *et al.* developed an oxygen self-sufficient fluorinated nanoplatform of pH-sensitive fluorocarbon-functionalized nanoparticles loaded with IR780, and used iRGD as a tumor-targeting and penetrating peptide 149. This oxygen self-sufficient nanoplatform could significantly enhance the tumor oxygenation and increased the generation of ^1^O_2_ *in vitro* and *in vivo*. These nanoplatforms, when injected into orthotopic breast cancer model, could remarkably inhibit the primary tumor growth and reduce lung and liver metastases. The aforementioned methods may suffer from premature oxygen release and storage issues. Furthermore, it has been reported that the stability of fluorinated nanoplatforms is essential. To enhance the stability of the nanoplatforms, Song *et al* developed lipid-polymer bilaminar oxygen nanobubbles with Ce6 conjugated to the polymer shell using a combination of emulsion-solvent evaporation and internal phase separation 150. The bilaminar shell of the nanobubbles rendered the nanobubbles biocompatible and stable. *In vitro* and *in vivo* results showed that nanobubbles exhibited much higher cellular uptake rates and tumor-targeting efficiency than free Ce6. A significant enhancement of photodynamic therapeutic efficacy was noticed due to the greatly enhanced ^1^O_2_ production powered by oxygen-encapsulated nanobubbles.

**Red blood cells** (RBC) are the primary oxygen source of our body tissues, carrying 270 million hemoglobin molecules. Due to the binding ability of each hemoglobin for up to four O_2_ molecules, RBCs have long been investigated for drug delivery. Moreover, natural RBC membranes could camouflage microparticles for successfully overcoming biological barriers with a long blood-circulation time. Taking advantage of these properties, Wang *et al.* developed RBC hybrid nanoplatforms, consisting of upconversion nanoparticles (UCNPs), and the rose bengal PS installation on the RBC surface 151. Under hypoxic conditions, the inactive hypoxia probe could trigger the O_2_ release from oxygenated hemoglobin under NIR excitation, further improving the PDT efficiency. Also, RBC nanoplatforms delayed uptake by the mononuclear phagocyte system by preferentially binding to the endothelium and decreasing retention in the reticuloendothelial system. Thus, RBC nanoplatforms show enhanced PDT under near-infrared irradiation to realize effective solid tumor eradication. Wan *et al.* prepared nanoscale RBC carriers containing sufficient oxyhemoglobin and gas-generating agent ammonium bicarbonate (ABC) for co-loading and controlled release of ICG and DOX 152. These nanoplatforms showed nearly identical PTT efficiency both *in vitro* and *in vivo*, but their PDT efficiency was improved significantly due to oxyhemoglobin. ABC was decomposed into NH_3_ and CO_2_, further triggering the rapid release of DOX exerting cytotoxic effects. The combination of enhanced PDT and released DOX significantly inhibited breast cancer cell growth in mice and induced cell apoptosis. *In vivo* experiments confirmed that the nanoplatforms almost completely ablated breast tumors and further suppressed tumor recurrence and metastasis.

**Figure 7:**
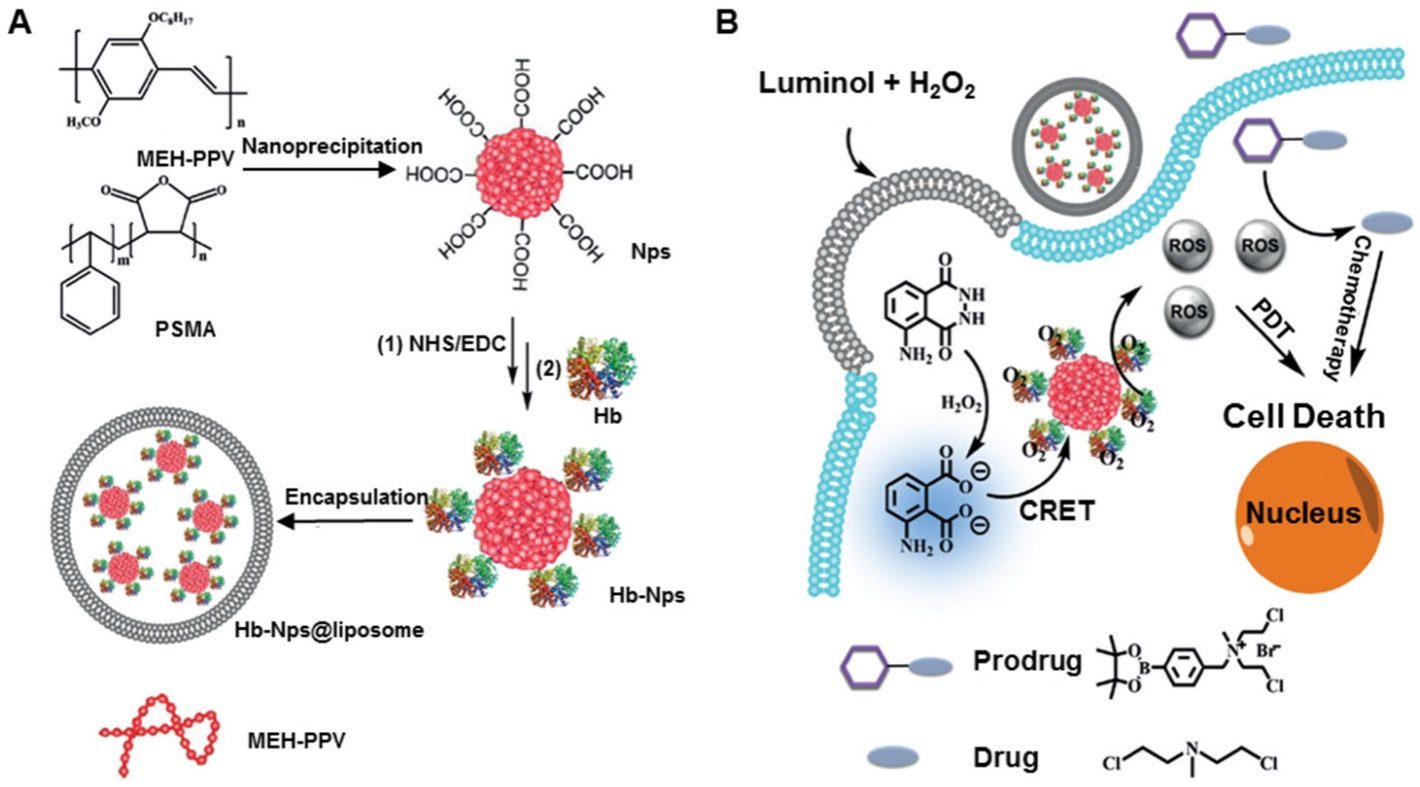
** A.** Schematic illustration of the preparation of Hb-NPs@liposome. **B.** the luminescing and oxygen-supplying system for phototherapy. Adapted with permission from ref. 154. Copyright 2019 Wiley-VCH.

**Hemoglobin** (Hb), the endogenous protein abundant in RBCs, has been used as an oxygen carrier for PDT. Compared to other carriers, hemoglobin is easily available and safe *in vivo*. However, the short circulation life, instability, and easy formation of dimers impair the treatment effect. To solve these problems, Wang *et al.* developed a hemoglobin polymer-conjugated nanoplatform as the carrier of both oxygen and the PS to enhance PDT 153. The amphiphilic triblock copolymer, poly (ethylene glycol) methyl ether-block-poly acrylic acid-block-polystyrene, was synthesized by atom transfer radical polymerization. The polymer and PS were covalently conjugated with Hb, and, compared with free Hb, the resulting nanoplatform showed a high tolerance to oxidation and trypsin digestion while retaining its O_2_ binding capacity. More importantly, the nanoplatform could readily generate ^1^O_2_ and kill 4T1 cells *in vitro* under light irradiation, exerting better phototoxicity with the oxygen supply of Hb. In another study, Jiang* et al.* prepared Hb-linked conjugated polymer nanoparticles, which were encapsulated in fusogenic liposomes (Hb-NPs@liposome) (Figure 7A) 154. Due to the catalyzed activation of luminol, the conjugated poly[2-methoxy-5-(2-ethylhexyloxy)-1,4-phenylenevinylene] (MEH-PPV) polymer, by the oxygen supplied by Hb, the nanoparticles could acquire chemiluminescence resonance energy transfer to produce ROS. This novel system did not require an external light source and circumvented the insufficient level of molecular oxygen for PDT. The luminescing and O_2_-supplying system offers the possibility of simultaneous PDT and chemotherapy (Figure 7B).

#### *In situ* generation of oxygen-nanoplatforms

**Figure 8:**
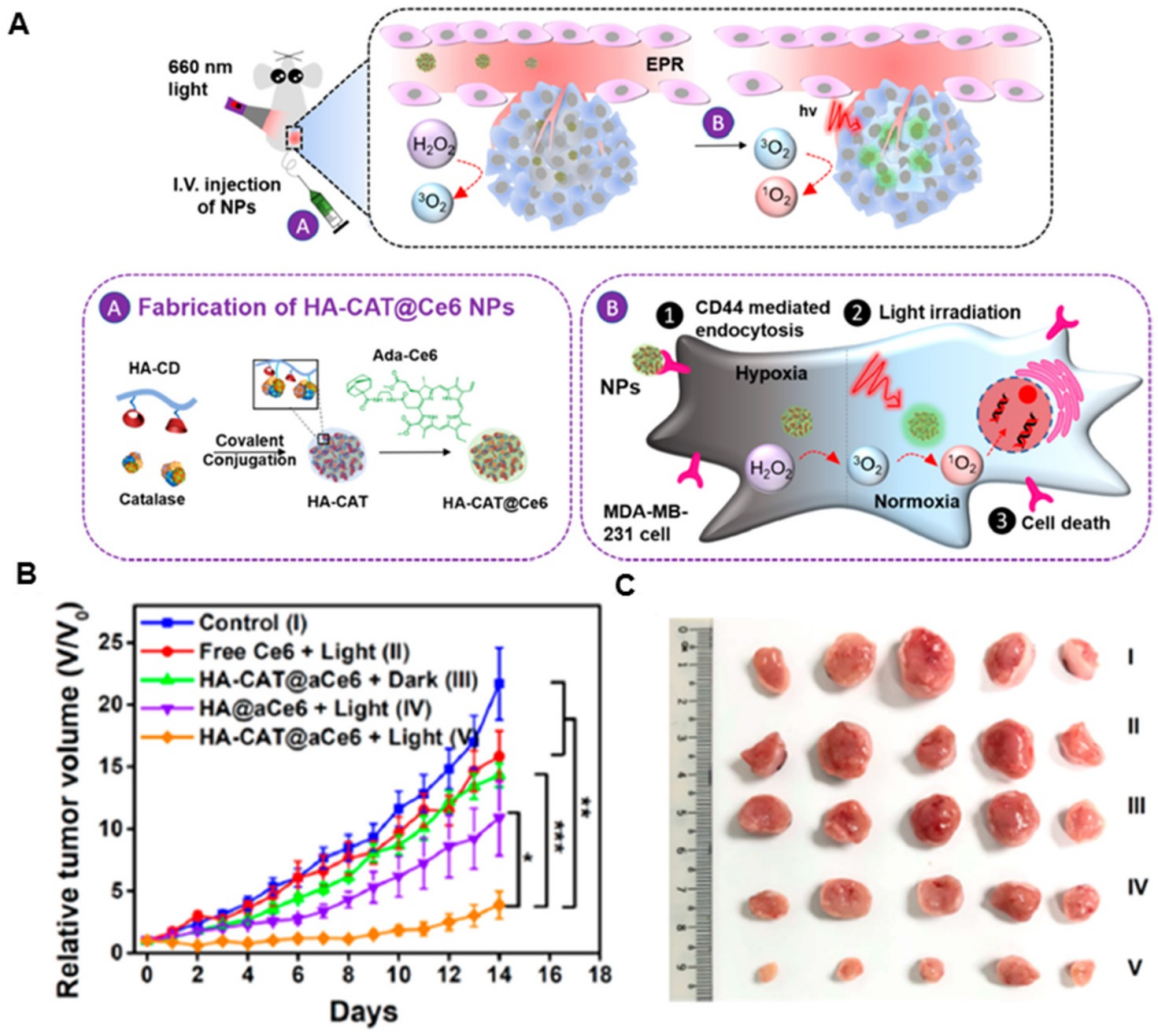
Catalase-integrated hyaluronic acid for enhanced PDT. **A.** Schematic illustration of the processes after intravenous injection of HA-CAT@aCe6 nanoplatforms into tumor-bearing mice. **B.** Relative tumor volumes of MDA-MB-231 tumor bearing mice with five treatment groups over 14 days. **C.** Photos of excised tumors from mice on the 14^th^ day after these treatments. Adapted with permission from ref. 163. Copyright 2019 American Chemical Society.

**Catalase** is a specific catalytic enzyme with an extremely high turnover to decompose H_2_O_2_ into O_2_ and has been explored in combination with other therapeutic approaches to construct several types of nanoplatforms to relieve tumor hypoxia 155-158. However, after systemic administration, catalase is unstable in the presence of proteases in blood circulation. Various methods, such as encapsulation of the enzyme into inorganic nanoparticles or polymeric capsules, have been designed to protect catalase from protease digestion 159, 160. Liu's group designed a catalase enzyme-encapsulated, Ce6-loaded hollow silica nanoplatform with rationally pH-responsive charge-convertible, mitochondria-targeting surface engineering 161. Within the acidic tumor microenvironment, the nanoplatform showed charge-conversion from negative to positive, accelerating cellular internalization and tumor retention. Also, the triphenylphosphine of this nanoplatform could enhance PDT by targeting mitochondria. Eventually, the nanoparticles could decompose endogenous H_2_O_2_ in the tumor and alleviate tumor hypoxia, greatly enhancing PDT treatment of solid tumors. Furthermore, the nanoparticles showed synergistic effects when combined with anti-PD-L1 checkpoint blockade to induce robust antitumor immunity, which could suppress tumor growth without direct light exposure. Subsequently, his group also loaded catalase into the capsule by in situ free-radical polymerization, using double-bonded meso-tetra(phydroxyphenyl)porphine as the cross-linker to enable condensed grafting of short polyethylene glycol chains on the protein surface as a permeable brush-like safeguard 162. The catalase-entrapped nanocapsules exhibited efficient passive retention in tumors after intravenous injection, which could significantly overcome tumor hypoxia by triggering the decomposition of endogenous tumor H_2_O_2_ into oxygen, achieving a remarkable antitumor therapeutic effect. Also, the nanocapusles showed greatly prolonged blood circulation, with the blood half-life of ≈6.42 h and effective tumor accumulation of 6.03 ± 1.78% ID g^-1^. However, it was reported that the relative enzymatic activity of catalase-encapsulated nanocarriers might decrease during the synthetic processes. To tackle the issue, Zhao's group developed therapeutic nanoplatforms consisting of catalase-encapsulated β-cyclodextrin-HA nanoparticles loaded with adamantane-modified Ce6 by supramolecular means (Figure 8A) 163. The obtained therapeutic nanoplatform could target overly expressed CD44 receptors on MDA-MB-231 cells, where the encapsulated catalase could decompose the endogenous H_2_O_2_ to generate O_2_ for alleviating hypoxia in cells incubated under hypoxic conditions. The nanoplatform also exhibited high cellular cytotoxicity under hypoxic condition because of the ability of catalase in mitigating hypoxia. *In vivo* experiments revealed that the tumor growth from group V was the most inhibited one among all groups, illustrating the highest antitumor efficacy of HA-CAT@aCe6 (Figure 8B and 8C).

**Figure 9:**
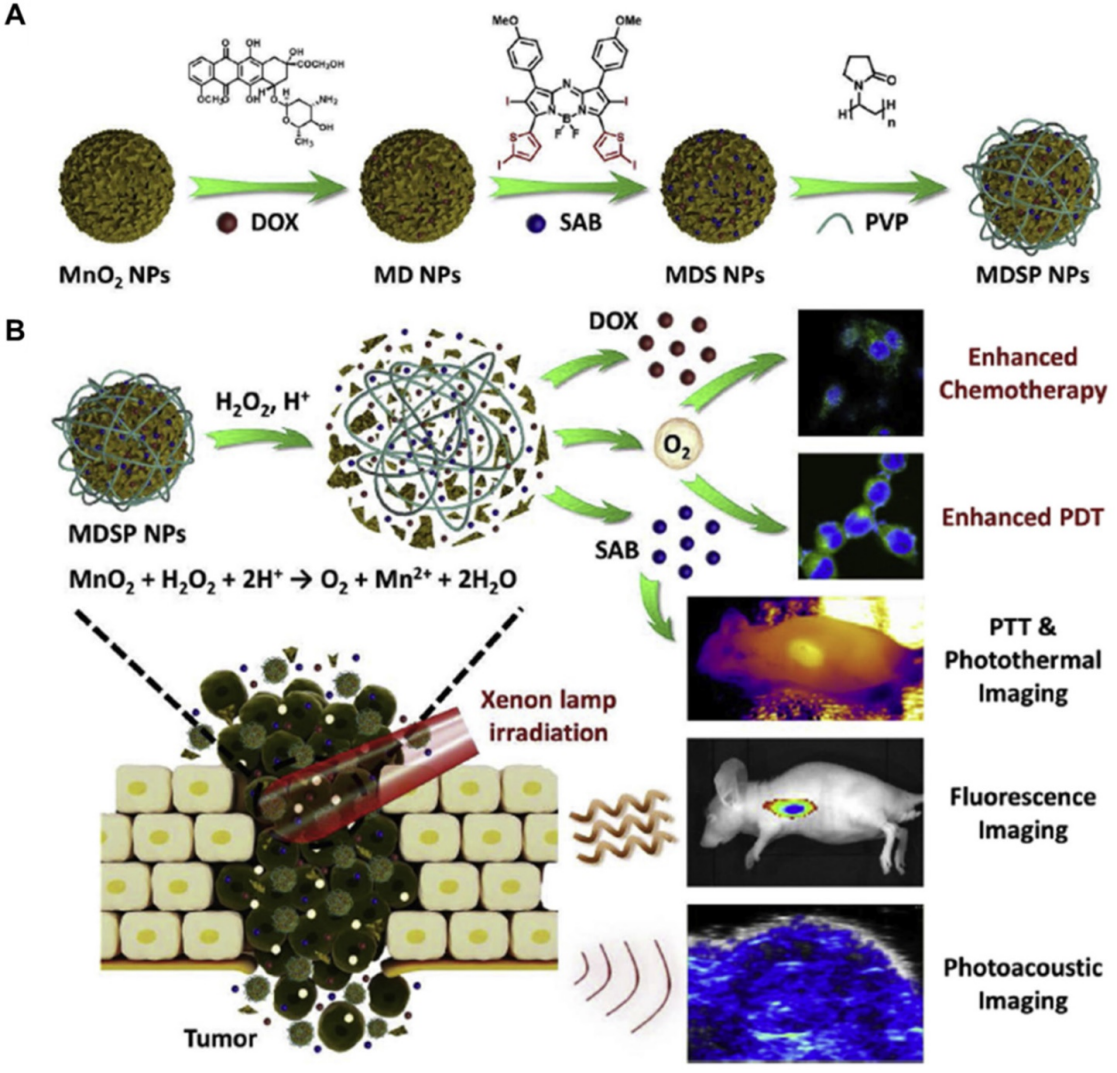
Hydrangea-structured nanoplatforms for tumor imaging and therapy.** A.** Schematic illustration of the fabrication of MDSP NPs. **B.** MDSP NPs for tumor microenvironment responsive chemo/photodynamic/photothermal therapy under xenon lamp irradiation. Adapted with permission from ref. 175. Copyright 2019 Elsevier.

**Manganese dioxide (MnO_2_)-**based nanoplatforms have attracted considerable interest in bio-applications. In response to the low pH tumor microenvironment, MnO_2_ nanoparticles can be rapidly broken up. Therefore, MnO_2_-based drug nanoplatforms show enhanced drug release in the acidic tumor microenvironment 164, 165. MnO_2_-based nanoplatforms could also catalyze H_2_O_2_ to produce O_2_ efficiently to improve tumor oxygenation *in vivo* 166-171. Zhu *et al.* developed multifunctional Ce6-loaded MnO_2_ nanoparticles with surface polyethylene glycol modification (Ce6@MnO_2_‐PEG) that could effectively strengthen the efficacy of PDT due to the increased intracellular O_2_ level generated from the reaction between MnO_2_ and H_2_O_2_^172^. *In vivo* results showed that Ce6@MnO_2_-PEG nanoparticles could accumulate in the tumor, and were gradually decomposed into Mn^2+^ ions to offer a strong T_1_ MR contrast. The tumor oxygenation level greatly increased due to MnO_2_-triggered O_2_ production from H_2_O_2_ within the tumor microenvironment. Compared with traditional PS approved by the Food and Drug Administration, aza-boron-dipyrromethene (aza-BODIPY) derivatives have exhibited excellent photophysical properties, such as near-infrared absorption, strong visible light molar absorption coefficient, and good light stability 173, 174. As shown in Figure 9A, Tang *et al* loaded aza-BODIPY photosensitizer and anticancer drug DOX onto the hydrangea-structured MnO_2_ nanoparticles, further modifying with amphiphilic polyvinylpyrrolidone to increase the physiological stability and biocompatibility 175. The resultant nanoplatform (MDSP) showed strong NIR absorption, significant photothermal effect, rapid degradation, and efficient oxygen-self-generation in the presence of H_2_O_2_. Especially, MnO_2_ could react with H_2_O_2_ and H^+^ in the tumor microenvironment to produce oxygen and overcome tumor hypoxia, enhancing the chemotherapeutic efficiency and PDT. As demonstrated by *in vivo* fluorescence and photoacoustic imaging, MDSP nanoparticles were preferentially accumulated at the tumor site, which could induce hyperthermia to alleviate hypoxia, promote the uptake of therapeutic nanoparticles, and further reduce the resistance to improve the therapeutic efficiency (Figure 9B). Besides, organic semiconductor PS is also widely studied. Zhu *et al.* developed near-infrared light excitable semiconducting hybrid nanoparticles of poly(cyclopentadithiophene-alt-benzothiadiazole) 176 in which the core served as the photodynamic agent for NIR fluorescence imaging, while the MnO_2_ nanosheets converted H_2_O_2_ to O_2_ in hypoxic and acidic tumor microenvironment. In contrast to the uncoated nanoparticles, the oxygenic nanoparticles generated 2.68-fold more ^1^O_2_ in the hypoxic and acidic conditions under NIR laser irradiation at 808 nm. Due to such an oxygen-generation property, the nanoplatforms could effectively eradicate cancer cells both *in vitro* and *in vivo*.

**Figure 10:**
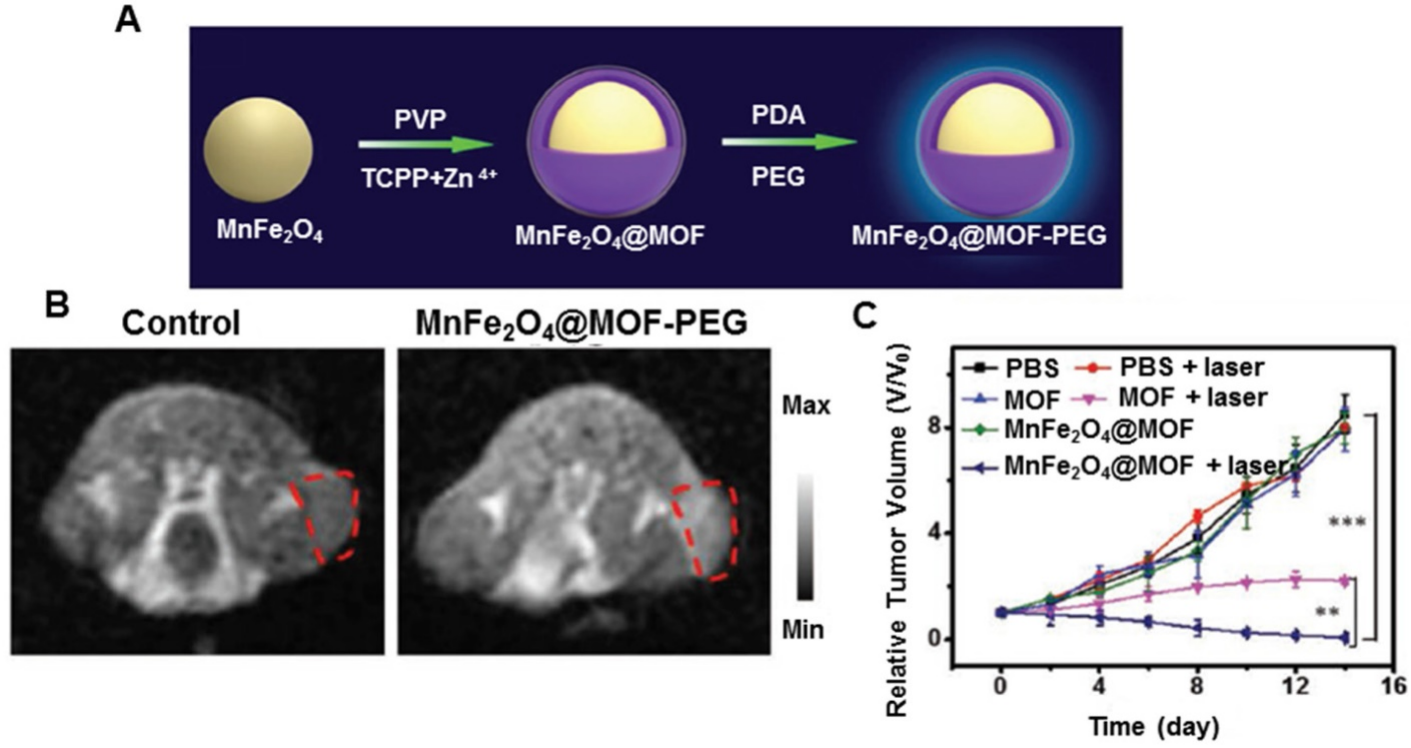
MnFe_2_O_4_@metal-organic frameworks for enhanced PDT. **A.** Schematic illustration of the synthesis process of MnFe_2_O_4_@MOF core-shell nanostructure. **B.** *In vivo T*_1_-weighted MR images of mice at 24 h post injection of PBS or MnFe_2_O_4_@MOF-PEG. **C.** Tumor growth curves of each group post-intratumoral administration under laser irradiation. Adapted with permission from ref. 179. Copyright 2019 Wiley-VCH.

Despite the use of MnO_2_ nanoparticles to generate O_2_ or consume GSH in the tumor microenvironment, several limitations, including rapid consumption of MnO_2_, transient O_2_ generation or GSH depletion, hamper the efficient regulation of tumor hypoxia and fail to continuously and synchronously enhance ROS-based therapeutic effect 177. Overcoming these drawbacks requires the development of a persistent strategy to regulate tumor microenvironment. To enhance PDT efficiency, Kim *et al.* developed biocompatible manganese ferrite nanoparticles anchored onto mesoporous silica nanoparticles 178. Exploiting the continuous generation of oxygen by MnFe_2_O_4_ nanoparticles through the Fenton reaction under physiological conditions, the nanoplatform could enhance ROS generation of PS, thus improving the therapeutic outcome of PDT for tumors *in vivo*. Furthermore, these nanoplatforms exhibited T_2_ contrast effect in MRI, allowing *in vivo* tracking of nanoplatforms. Based on previous findings, Zhang *et al.* developed biocompatible nanoplatforms by concentrating a coating of porphyrin-based MOF as the PS and MnFe_2_O_4_ as the nanoenzyme (Figure 10A) 179. The MnFe_2_O_4_@MOF nanoplatform exhibited both catalase and glutathione peroxidase-like activities. Once internalized in the tumor, the nanoparticles could continuously catalyze H_2_O_2_ to produce O_2_ by cyclic Fenton reaction and also persistently reduce GSH in the presence of H_2_O_2_, decreasing the depletion of ROS upon laser irradiation during PDT. MnFe_2_O_4_@MOF was also used for MRI-guided precision cancer therapy (Figure 10B). An enhanced photodynamic therapeutic effect arose from simultaneously self-producing O_2_ and self-decreasing GSH (Figure 10C).

**Figure 11:**
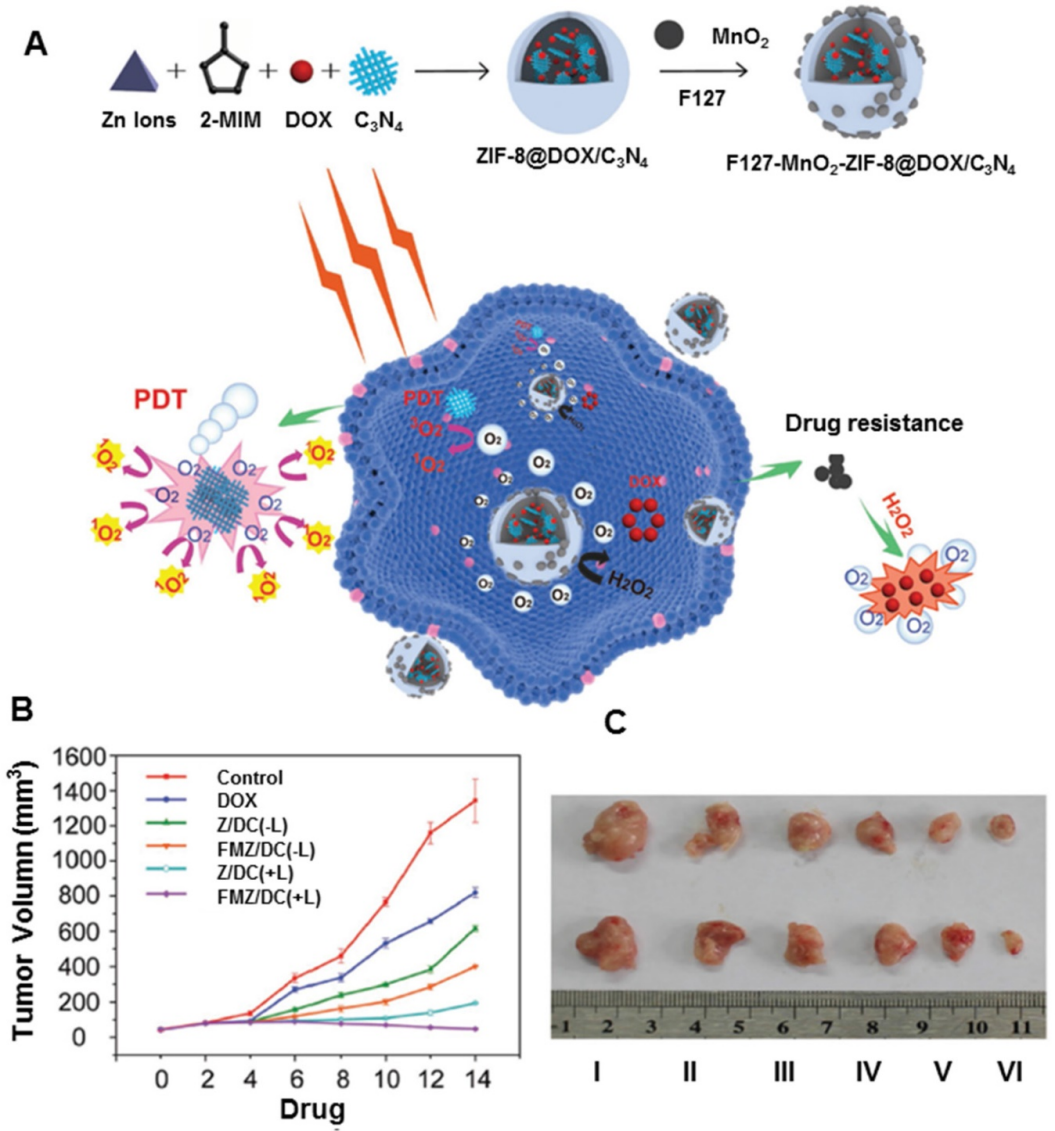
MnO_2_ nanodots-anchored nanoplatforms for chemo-PDT. **A.** Schematic illustration of the fabrication of FMZ/DC nanoplatforms with oxygen generation enhancing the chemo-photodynamic therapy. **B.** Tumor growth curves of different groups of 4T1 tumor-bearing mice. **C.** Images of tumors collected from different groups of mice 14 day after different treatment. Adapted with permission from ref. 181. Copyright 2018 Wiley-VCH.

Besides MnO_2_ carriers for PDT, hybrid nanoplatforms assembled from MnO_2_ and other nanomaterials are sometimes more effective in enhancing PDT. Bhattacharyya *et al.* developed unique two-dimensional hybrid nanoplatforms based on PEGylation of MnO_2_-decorated p-MoS_2_/n-rGO heterojunction nanosheets 180. The resulting nanoplatforms showed better biocompatibility and colloidal stability in physiological solutions. The p-n heterojunction directed NIR-triggered generation and separation of electron-hole pairs, which improved the production of ROS via photocatalysis. Also, MnO_2_ increased intracellular O_2_ by reacting with endogenous H_2_O_2_ in the cellular microenvironment. The hybrid nanoplatforms have been demonstrated as PDT-enhanced agents for cancer therapy. In another study, Wang *et al.* developed a chemo-photodynamic nanoplatform (FMZ/DC) by one-pot encapsulation of g-C_3_N_4_ and DOX in ZIF-8, then loading MnO_2_ nanodots and surface-modifying F127 (Figure 11A) 181. The hybrid nanoplatforms could generate oxygen upon the addition of H_2_O_2_, further demonstrating the enhanced therapeutic effects for both chemotherapy and PDT therapy. *In vivo* tumors treated with FMZ/DC began to shrink after 2 days of chemo-photodynamic therapy. The outstanding therapeutic effect could be ascribed to the oxygen-generating ability of the nanoplatform, improving the efficiency of photodynamic and chemotherapeutic treatments (Figure 11B and 11C).

**Figure 12:**
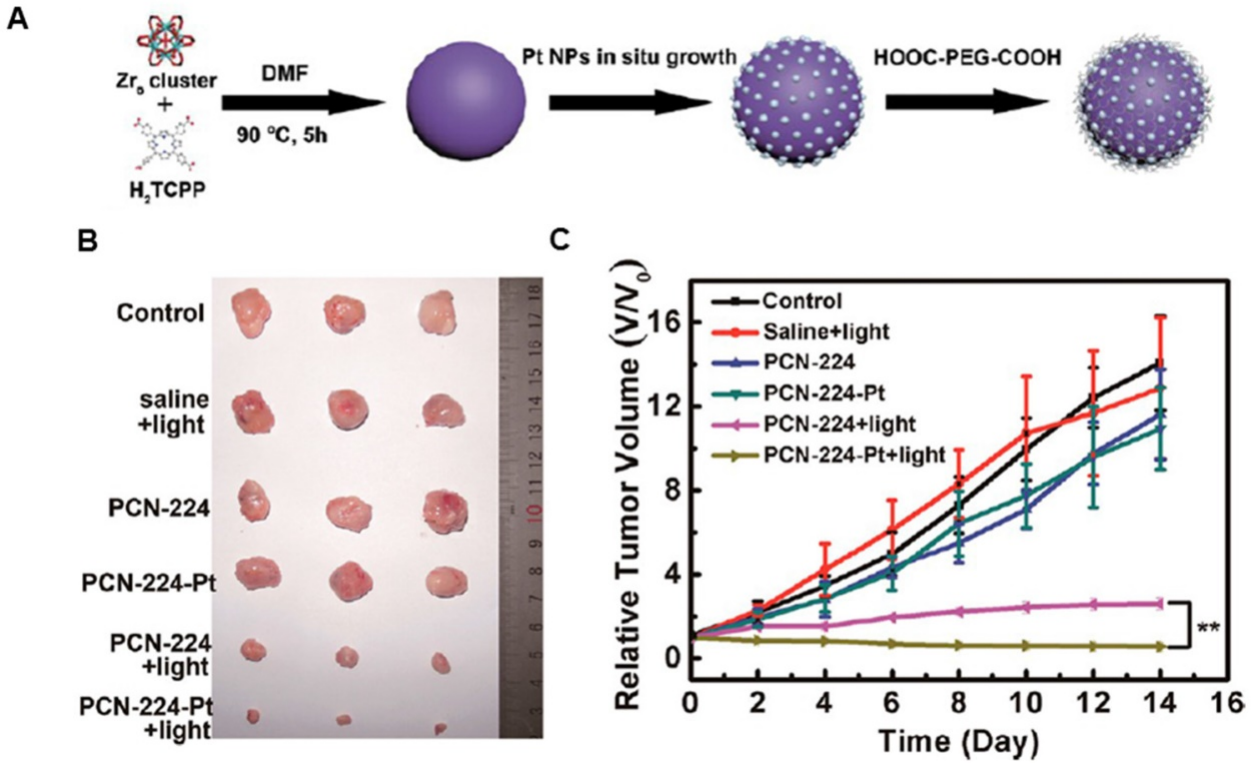
Nanozyme decorated MOF for enhanced PDT. **A.** Schematic illustration of the preparation process of PCN-224-Pt. **B.** Representative photographs of the tumor dissection. **C.** Relative tumor volume after various treatments indicated. Adapted with permission from ref. 185. Copyright 2018 American Chemical Society.

Encouraged by the excellent catalytic performance of natural enzymes, varieties of synthetic structures have been designed to mimic the functions and complexities of natural enzymes over the past few decades. Various artificial nanozymes are emerging and attracting extensive research interest 182, 183. Platinum nanoparticles are well-known catalysts for many chemical reactions, whose enzyme mimetic activities have been reported previously 184. As illustrated in Figure 12A, Zhang *et al.* developed a porphyrinic Zr-MOF derived from PS and Zr in which Pt nanoparticles were homogeneously decorated on MOF via an *in-situ* reduction and further coated with PEG to greatly improve the biocompatibility and physiological stability 185. The obtained nanoplatform with high catalase-like activity induced the decomposition of H_2_O_2_ to produce O_2_ at the hypoxic tumor site, facilitating the formation of cytotoxic ^1^O_2_ to kill cells. The tumor growth was completely inhibited when the mice were injected with the nanoplatform (PCN-224) after irradiation treatment (Figure 12B and 12C). Thus, the nanoplatform exhibited much improved PDT efficiency via H_2_O_2_-activated generation of O_2_ and light-irradiated formation of ^1^O_2_. Similarly, Wei *et al.* designed Pd@Pt bimetallic nanoplates, which were further covalently conjugated with Ce6 and bifunctional PEG 186. The nanoplatform could produce O_2_ over a long period by reacting with H_2_O_2_ in the tumor sites. Also, the moderate photothermal effect of the nanoplatform under 808 nm laser irradiation could further improve the PDT efficacy in hypoxic tumors by accelerating its catalytic decomposition of H_2_O_2_. Both *in vitro* and *in vivo* results indicated that the nanoplatform effectively delivered PS to cancer cells/tumor sites and triggered the decomposition of endogenous H_2_O_2_ to generate oxygen, resulting in a remarkably enhanced PDT efficacy.

#### Other nanoplatforms for generating oxygen

Besides the nanoplatforms mentioned earlier, oxygen bonding and in situ generation of oxygen, some researchers have attempted to endow nanoplatforms with oxygen by other means to enhance PDT. Wang *et al.* developed core-shell nanoparticles based on a double emulsion method, wherein the H_2_O_2_/ poly(vinylpyrrolidone) complex and poly(lactic-co-glycolic acid) (PLGA) served as the core and shell, respectively. The hydrophilic H_2_O_2_/poly(vinylpyrrolidone) complex acted as an oxygen source, while hydrophobic IR780 was the PTT/PDT agent. When the resultant nanoplatforms were internalized by HepG2 cells, they could generate the photothermal effect where ROS was released to kill cancer cells under an 808 nm laser irradiation. Moreover, the encapsulated H_2_O_2_ could supply additional oxygen and, in turn, significantly enhance the PDT effect 187. The nanoplatforms accumulated in the xenograft tumor and could inhibit tumor growth due to combinational PTT and enhanced PDT upon NIR light irradiation. Xie *et al.* developed an O_2_-loaded pH-responsive multifunctional nanoplatform (UC@mSiO_2_-RB@ZIF-O_2_-DOX-PEGFA) with enhanced chemo-photodynamic therapeutic effect 188. NaYF_4_:Yb/Er@NaYbF_4_:Nd@NaGdF_4_ nanoparticles were employed as both upconversion/MRI matrix and motivator for rose red PS in PDT with deep penetration depth. Zeolitic imidazolate framework-90 was capped outside of mSiO_2_ as an O_2_ reservoir to quickly release O_2_ in the tumor microenvironment, thereby improving the PDT efficiency. Additionally, DOX and NH_2_-poly(ethylene glycol)-modified folic acid were covalently adsorbed on the surface of nanoparticles for synergetic therapy. Li *et al.* developed upconversion nanophotosensitizers with hyperbaric oxygen (HBO) to change the extracellular matrix for enhanced photodynamic cancer therapy 189. The UCNPs were developed for Nd^3+^-sensitized sandwiched structure, wherein the upconversion core served as a light transducer to transfer energy to the neighboring rose bengal to produce ROS. With HBO, the photodynamic process could induce abundant ROS in the intrinsically hypoxic tumor. Furthermore, HBO-assisted PDT decomposed collagen in the extracellular matrix of tumors and facilitated the diffusion of oxygen and penetration of nanoplatforms into the deeper area of the tumor. Such a synergic effect eventually caused enhanced therapeutic efficacy at a low laser power density as compared with those using UCNPs alone.

### Water-splitting materials

Due to possibly worsening hypoxia, the PDT may cause irreversible tumor metastasis or drug resistance. Highly efficient production of O_2_ can potentially compensate for the tumor hypoxic microenvironment. Compared to the aforementioned nanoplatforms able to produce oxygen, water is the most abundant compound in the physiological environment 190 and could provide unlimited raw material for O_2_ generation. To date, a variety of splitting materials have been innovatively constructed to generate oxygen and hydrogen for clean, renewable resources, also extending the strategy to PDT 191. The commonly used water-splitting nanoplatforms include oxides (titanium dioxide), carbon or nitrogen compounds (carbon nitride and MXene), and sulfides.

**Titanium dioxide (TiO_2_)** has received considerable attention for its adjustable band gap, band position, superior photostability, high intrinsic catalytic activity, abundance, low toxicity, and inexpensiveness 192-194. Yang *et al* developed carbon-nanodot-decorated TiO_2_ nanotubes as a nanoplatform for PDT 195. In this formulation, carbon dots (CDots) could increase the light absorption response and narrow the band-gap compared with anatase TiO_2_ nanoparticles. Interestingly, CDots/TiO_2_ nanotubes could absorb the 650 nm NIR light, where the emission wavelengths (325-425 nm) of CDots excite TiO_2_ nanotubes to form electron/hole (e^-^/h^+^) pairs, inducing the reaction with the adsorbed oxidants to produce ROS. Besides, the CDots showed high chemical catalytic activity for H_2_O_2_ decomposition. The excellent PDT performance actuated by 650 nm light was verified by *in vivo* assays. Using an organometallic ruthenium complex (N_3_) as a conjugator, Gilson *et al* prepared TiO_2_-N_3_ nanoplatforms 196. Upon exposure of TiO_2_-N_3_ to light, N_3_ injected electrons into TiO_2_ to produce three- and four-fold hydroxyl radicals and hydrogen peroxide. Furthermore, the inability of TiO_2_ to produce H_2_O_2_ under hypoxic conditions suggested that N_3_ facilitated the depletion of residual H_2_O_2_ and rapid conversion of the low oxygen concentration into •OH, thus increasing the concentration of this species. TiO_2_-N_3_ maintained three-fold higher hydroxyl radicals than TiO_2_ under hypoxic conditions via N_3_-facilitated electron-hole reduction of adsorbed water molecules. These results demonstrated a mechanism to convert and maintain cytotoxic •OH production by harnessing the reductive power of ruthenium complexes, which efficiently reduced low levels of oxygen for ROS production.

**Figure 13:**
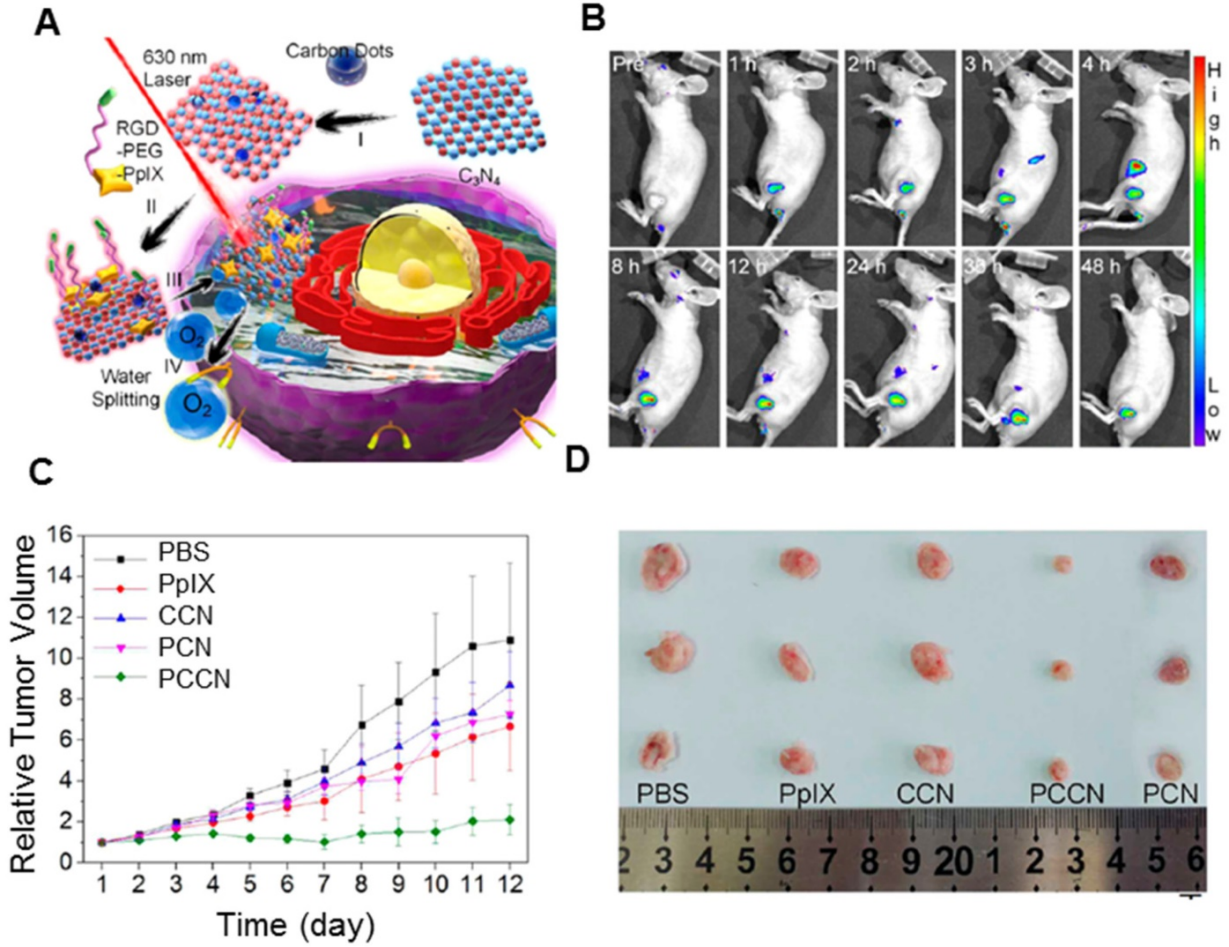
** A.** Structure of PCCN and schematic diagram of 630 nm light-driven water splitting enhanced PDT. **B.** *In vivo* fluorescence imaging of PCCN at different time points after intravenous injection. **C.** Tumor images at the 12^th^ day post-treatment. **D.** Relative tumor volume post-treatment, the intravenous injection of samples was performed on different days. Adapted with permission from ref. 199. Copyright 2016 American Chemical Society.

**Carbon nitride** (C_3_N_4_) has received considerable attention for the adjustable band gap and band position 197, 198. After the modification, water splitting can be driven under high penetrable red light >600 nm, which makes C_3_N_4_ suitable for *in vivo* therapy. Most importantly, due to the absence of metal elements, C_3_N_4_ is thought to be a highly biocompatible material for biomedical applications. As presented in Figure 13A, Zheng *et al.* developed CDots-doped C_3_N_4_ nanoparticles, and further modified with an amphipathic polymer (PpIX-PEG-RGD) 199. The as-prepared nanoplatform could split water to generate O_2_ under 630 nm laser radiation with high efficiency. The PS could further transmit the energy to the produced O_2_ to generate cytotoxic ^1^O_2_. *In vitro* study showed the nanoplatform could increase the intracellular O_2_ concentration and improve the ROS generation in both hypoxic and normoxic environments. After the injection, PpIX fluorescence started to accumulate in the tumor (Figure 13B). The *in vivo* experiment indicated that CDots-doped C_3_N_4_ (CCN) nanoparticles and PpIX showed little inhibitory effects on 4T1 tumors. However, treatment with polymer-modified, CDots-doped carbon nitride nanoparticles (PCCN) exhibited remarkable growth inhibition against tumors with only one injection (Figure 13C and 13D), which could be attributed to its tumor targeting and O_2_ generation. Recent studies also reported that metal ion-doped C_3_N_4_ nanoplatforms showed better-enhanced PDT. Jiang *et al.* developed the fusiform Fe^III^-doped C_3_N_4_ nanoplatform, further modified by mitochondria-targeting molecular (4-carboxybutyl) triphenylphosphonium bromide (TPP) 200. The ultrahigh surface area of the nanoplatform enhanced the loading capacity of methylene blue, while mitochondria-targeting TPP agent improved the ROS concentration, thus accelerating the mitochondrion dysfunction and further triggering cell death during PDT. The doping of Fe^III^ showed excellent catalytic performance towards H_2_O_2_ in cancer cells to generate O_2_, thus overcoming tumor hypoxia and enhancing the PDT efficacy. In addition, the introduction of Fe^III^ endowed the Fe^III^-doped C_3_N_4_ nanoplatform with an effective T_1_-weighted MRI contrast ability for simultaneous imaging and therapy. Likewise, Qu *et al.* reported that the combination of Cu^2+^ and g-C_3_N_4_ nanosheets (Cu^2+^-g-C_3_N_4_) led to increased light-triggered ROS generation as well as the depletion of intracellular GSH levels 201. Consequently, the ROS generated under light irradiation could be depleted less by reduced GSH, thus improving efficiency. Importantly, Cu^2+^-g-C_3_N_4_ nanosheets could catalyze the reduction of molecular oxygen to the superoxide anion or hydrogen peroxide to the hydroxyl radical, both of which accelerated the generation of ROS. This synergy of improved ROS generation and GSH depletion could enhance the efficiency of PDT for cancer therapy.

Wang *et al.* developed a biomimetic ultrathin graphdiyne oxide from oxidized and exfoliated graphdiyne, which was further modified by iRGD peptide-modified RBC membrane 202. The obtained nanoplatform showed prolonged blood circulation via RBC membrane camouflage along with enhanced extravascular and hypoxic region penetration by a functional iRGD peptide. The nanoparticles could efficiently catalyze water oxidation to release O_2_ and generate ^1^O_2_ using 660 nm irradiation. More importantly, the hyperthermia effect of ultrathin graphdiyne oxide could conveniently cause dilation of vessels and blood perfusion for overcoming perfusion-limited hypoxia. Consequently, with O_2_ evolving from photocatalysis water splitting together with blood perfusion from photothermal conversion, this nanoplatform alleviated diffusion- and perfusion-limited hypoxia synchronously and further enhanced PDT.

The above-mentioned results indicated that numerous materials could improve intratumoral O_2_ and ROS supplementation for enhanced PDT (Table 2). However, the limited O_2_ binding sites in Hb and low solubility of O_2_ in perfluorocarbon restricted the efficacy. Additionally, the low intracellular concentration of H_2_O_2_ significantly limited the O_2_ production yield and thus achieved only moderate efficacy in cancer therapy. As the most abundant compound in living organisms, water provides enough O_2_ for *in vivo* PDT. Therefore, the construction of highly catalytic nanoplatforms is essential for PDT.

## Physical effects of nanoplatforms for enhanced PDT

The efficiency of PDT is also affected by physical factors, such as light sources 203, magnetic field 204, electric fields 205, microwaves 206, and ultrasound 207. Compared with light, the microwave is a superior energy source to trigger the ROS generation for tumor therapy. However, microwave energy is considered to be insufficient for inducing free radical generation, making microwave dynamic therapy subject to certain restrictions 208. Ultrasound can activate PS for tumor cell destruction useful for penetrating deep tissues. This novel approach is known as sonodynamic therapy 209. Some studies have shown that the efficiency of ultrasound-activated PS needs to be further improved. The microwave of the electromagnetic spectrum has been extensively explored for tumor ablation in clinical settings, especially microwave thermal therapy because of the penetration depth in tissues, high heating efficiency, and negligible side effects. Magnetic nanoparticles are promising candidates for controlled drug delivery, hyperthermia therapy, and MRI 210. When subjected to oscillating magnetic fields, the magnetic nanoplatforms could generate heat for targeted tumor therapy. Moreover, magnetic nanoplatforms can absorb NIR light to induce hyperthermia. Therefore, a combination of magnetic nanoparticles and the PS in nanoplatforms leads to enhanced PDT efficacy. Some physical effects to enhance PDT are summarized in the Table 3.

### X-rays nanoplatforms

**Figure 14:**
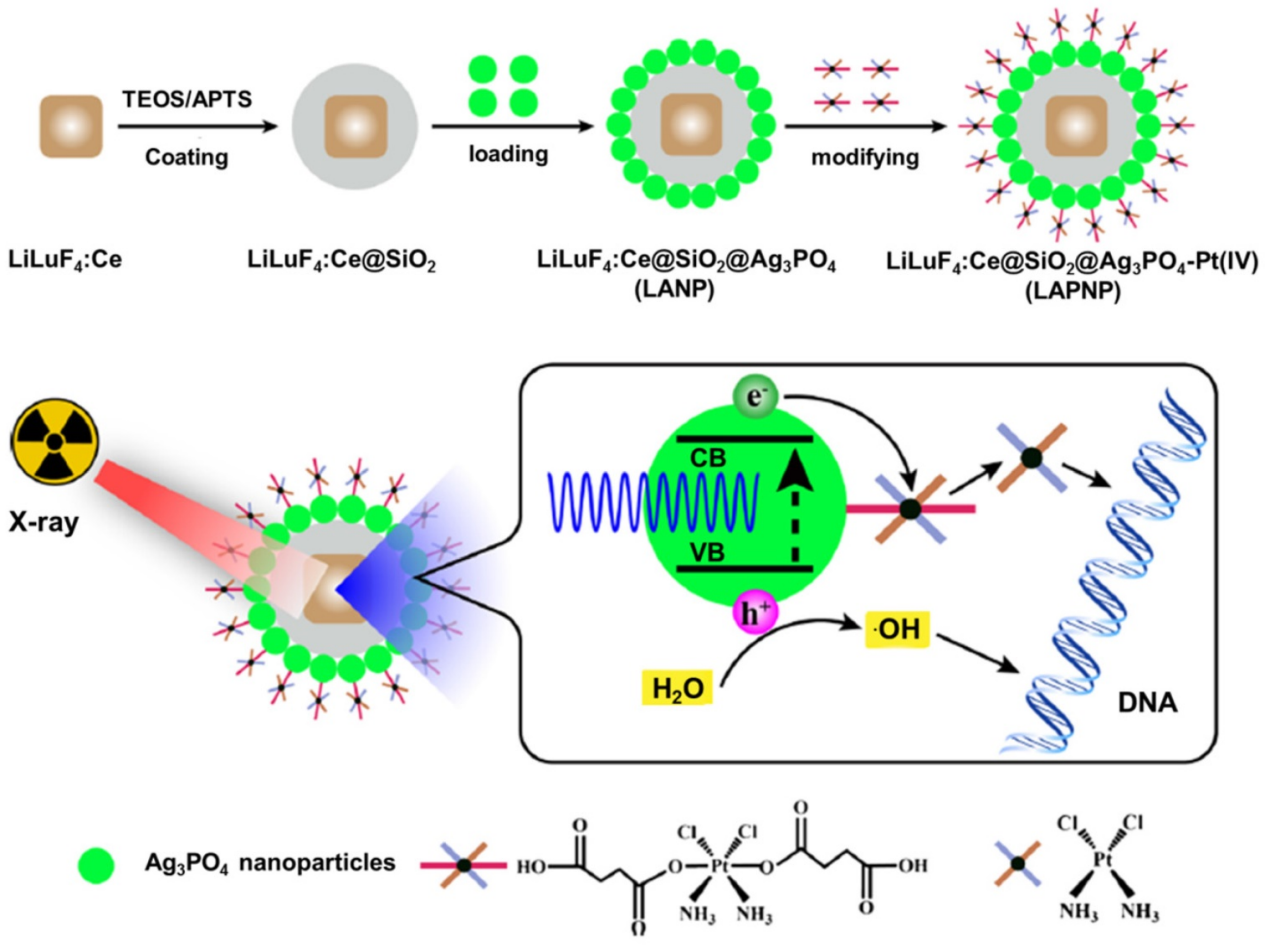
Illustration showing the preparation process and mechanisms underlying the effects of X-PDT with LiLuF_4_:Ce@SiO_2_@Ag_3_PO_4_@Pt(IV) nanoparticles (LAPNP). Adapted with permission from ref. 218. Copyright 2018 American Chemical Society.

Most of the solid tumors are located several millimeters under human skin, whereas, the penetration depth of UV/visible light into biological tissues is merely several micrometers 211. Such limited penetration depth results in low tumor-killing efficiency. Many efforts have been devoted toward the development of the novel nanoplatforms to surmount the challenges from such poor and limited light tissue penetration. These nanoplatforms, one the one hand, involve the introduction of enhanced chemi- or bioluminescent probes as well as multi-photon and upconverting materials. On the other hand, they utilize external ionizing radiation to penetrate deep into the human body as a neoadjuvant treatment. As is widely accepted, X-rays exhibit a high penetration depth in the human body for clinical imaging. It holds the potential to become an ideal excitation source for activating PS accumulated in deep tumor tissue 212, 213. In a typical X-rays assisted PDT, the absorbed X-ray energy was converted into photons with the appropriate wavelength, which were further absorbed effectively by PS. The resultant ROS from PS could directly damage the tumor cells, and the absorbed ionizing radiation could also generate radical species and break DNA double-strands 214, 215. To match the energy difference between X-rays (keV-MeV) and the light absorbed by PS (eV), lanthanide-based scintillators and heavy metals have been adopted to absorb X-rays and transmit the energy to PS, leading to efficient luminescence in the visible light region. Since Tb^3+^ shows efficient green luminescence that matches well with the absorption band of porphyrin. Chen's group synthesized water-soluble LaF_3_:Tb^3+^-porphin conjugates to study ^1^O_2_ generation under X-rays irradiation. Anthracenedipropionic acid (ADPA) was used to measure the ^1^O_2_ generated by the porphin and LaF_3_:Tb^3+^-porphin conjugates. Upon X-rays irradiation, the luminescence of ADPA quenched much faster in LaF_3_:Tb^3+^-porphin conjugates than in the porphin solution. Therefore, high production of ^1^O_2_ in the LaF_3_:Tb^3+^-porphin conjugates was verified because of the energy transfer from the LaF_3_:Tb^3+^ nanoparticles to porphin 216. Similarly*,* mesoporous LaF_3_:Tb^3+^ nanoparticles were proposed to load rose bengal for establishing an efficient fluorescence resonance energy transfer system 217. These results indicated that a large amount of ^1^O_2_ could be generated in X-rays PDT for deep-seated tumor treatment. Moreover, recent years have witnessed the development of* in vivo* applications of X-rays PDT. Wang *et al* developed hybrid nanoplatforms based on LiLuF_4_:Ce@SiO_2_@Ag_3_PO_4_@Pt(IV) to enhance the curative effects of X-rays PDT (Figure 14). LiLuF_4_:Ce nanoparticles were employed as the scintillator, and a cisplatin prodrug Pt(IV) was used as a sacrificial electron acceptor to increase the yield of hydroxyl radicals by boosting the separation of electrons and holes in Ag_3_PO_4_. Cisplatin was produced when Pt(IV) accepted electrons, which could damage the deoxyribonucleic acid in cancer cells, thereby enhancing the effect of X-rays PDT 218. To endow X-rays PDT with diagnosis information, enriched scintillation nanoparticles (CeF_3_:Gd^3+^,Tb^3+^), coated with mesoporous silica for loading rose bengal, were developed in the treatment of deep-seated tumors 219. The results showed an efficient tumor regression with synergistic nonradioactive radio-/photodynamic therapy under the guidance of computed tomography (CT) and MRI* in vivo*. Besides, other nanoplatforms of mesoporous silica-coated gold nanorods with conjugated europium (Eu) complexes were proposed for photothermal and X-rays PDT. Europium (Eu) complexes efficiently transfer the X-rays energy to hematoporphyrin for PDT treatment. *In vitro* and *in vivo* studies indicated that nanoplatforms showed CT and photoacoustic imaging for deep tumor penetration. An effective suppression of tumor progression was verified under X-rays and laser irradiation *in vivo* 220. Besides, QDs-based, silicon-based, and metal-based nanoscintillator have also been proposed and studied 221. In view of size-dependent photoluminescence of QDs and silicon semiconductor nanoparticles, their absorbance and photoluminescence could easily be adjusted for PDT. Moreover, they showed well biodistribution properties and could be eliminated from the body in a relatively short time. Remarkably, QDs could transfer energy to a molecular PS or directly to molecular O_2_, both of which can generate ^1^O_2_ for X-rays PDT. As metal-based nanoscintillators, gold nanoparticles exhibited excellent absorbers of X-rays and have been studied as radiosensitizers for radiotherapy. Deng and co-authors developed novel mitochondria targeted PLGA-based nanoplatforms containing ultrasmall gold nanoparticles and verteporfin. The nanoplatforms demonstrated a higher ability to generate ^1^O_2_ compared to PLGA-loaded verteporfin. Furthermore, they produced a large amount of ^1^O_2_ within the mitochondria *in vitro* upon X-rays radiation, thus triggering mitochondria-related apoptosis of cancer cells. Obviously inhibited growth of *in vivo* tumor with only a fraction of radiotherapy dose by X-rays PDT 222*.* Despite some progress have been made, some *in vivo* studies are conducted by intratumoral injection of nanoplatforms, which is ineffective to the non-invasive clinical treatment of deep-seated tumor due to poor specificity upon their uptake in these cancer cells. What's more, the interplay between PDT and ionizing irradiation remain unclear, and the low-dose irradiation, energy conversion as well as safety profiles need further explorations.

### Surface plasmon resonance of nanoplatforms

The electromagnetic-near-field enhancement mechanism of plasmonic nanoparticles has been exploited to control ^1^O_2_ generation. When the localized surface plasmon resonance (SPR) band was close to the absorption band of nearby PS, the absorption coefficient of PS could be significantly improved by the localized electric field, thereby increasing the production efficiency of ROS 223, 224. So far, plasmonic gold-based nanoplatforms have been explored to enhance the ^1^O_2_ by strong and tunable SPR. Li *et al.* developed a nanoplatform composed of mesoporous silica-coated gold nanorods incorporating indocyanine green 225. The SPR of gold nanorods was regulated to overlap with ICG exciton absorption. Such an overlap greatly increased the ^1^O_2_ yield of incorporated ICG under laser excitation. Furthermore, the nanoplatform possessed a synergistic PTT effect based on both Au nanorod and ICG. The integrated strategy significantly improved photodynamic destruction of breast tumor cells and inhibited the growth of orthotopic breast tumors in mice through the synergistic effect of PDT and PTT. To exploit the SPR advantages of gold nanorods, the nanoplatform consisting of NaYF_4_:Yb/Er conjugated with gold nanorods was prepared to boost the therapeutic efficiency of PDT 226. Methylene blue was embedded inside the silica shell to increase photostability and prevent drug leakage. UCNPs could convert NIR light to visible light to excite methylene blue for the generation of ROS. More importantly, gold nanorods could effectively increase upconversion efficiency and ROS content through a localized SPR effect. The mechanism of plasmon-enhanced PDT was verified by enhancing upconversion luminescence intensity through the plasmonic field and by increasing the light-harvesting capability and absorption cross-section of the system. Consequently, the nanoplatform could produce ROS and undergo efficient PDT *in vitro* and *in vivo*. The plasmonic Au nanoparticles integrated with the black phosphorus nanosheet simultaneously enhanced the ^1^O_2_ generation and hyperthermia by localized SPR effects for cancer phototherapy 227. The hybrid nanoplatform not only significantly increased 3.9-fold ^1^O_2_ production of black phosphorus nanosheet, but also achieved 1.7-fold increase in photothermal conversion efficiency upon single laser irradiation with 670 nm. When mice were subjected to laser irradiation, the nanoplatform showed stronger inhibition of tumor growth than other controls. The nanoplatform could almost completely restrain the tumor growth over 14 days due to enhanced PDT/PTT synergistic therapeutic effects.

### Other nanoplatforms through resonance energy transfer

**Figure 15:**
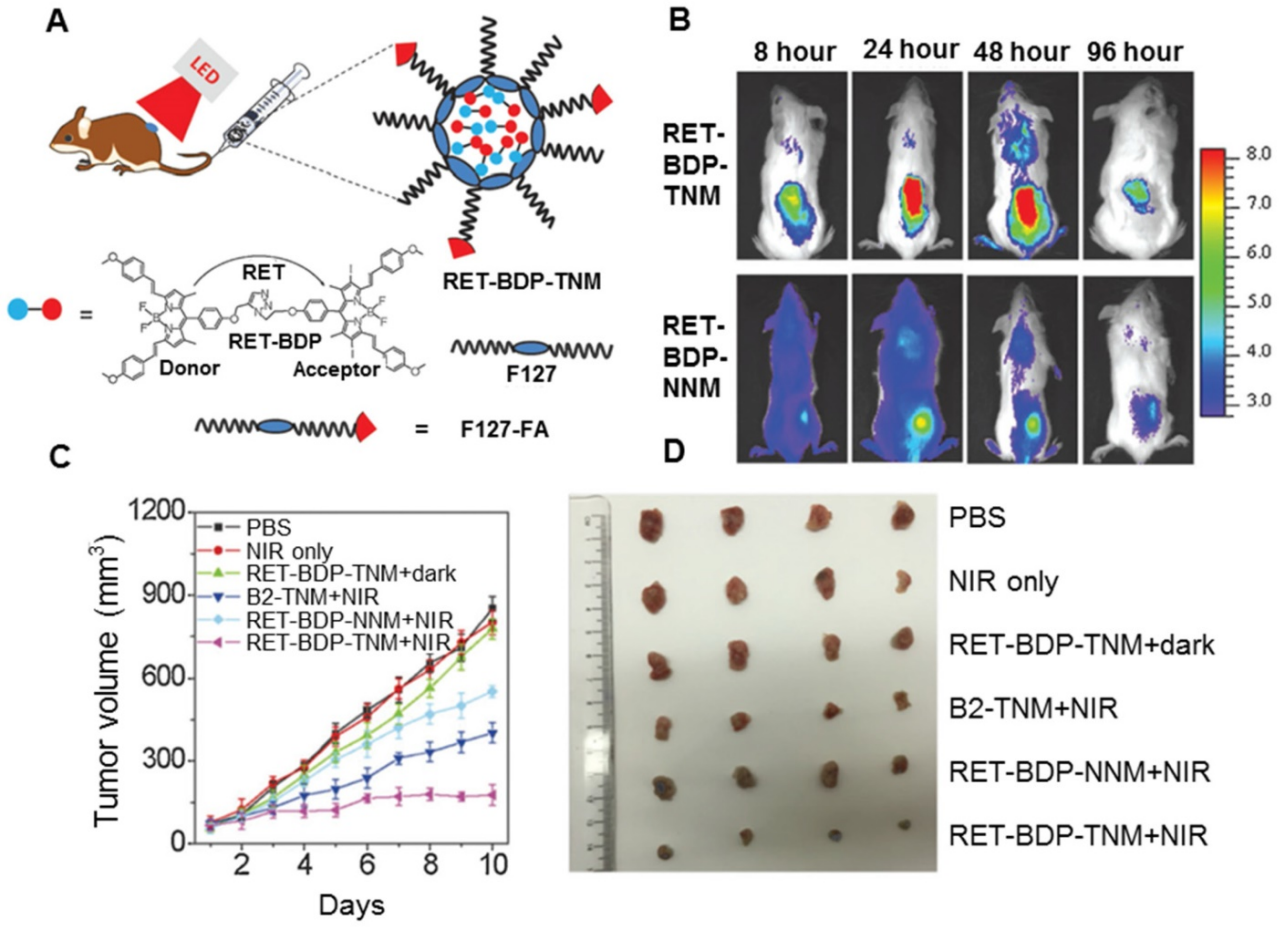
NIR photosensitized nanoparticles for enhanced PDT. **A.** Schematic illustration of RET-photosensitizer-mediated PDT. **B.** Specific targeted NIR-fluorescence tumor imaging *in vivo.*** C.** Tumor growth inhibition by RET-BDP-TNM-mediated PDT in 4T1 tumors. **D.** Digital photos of tumors for the four groups of mice. Adapted with permission from ref. 234. Copyright 2017 Wiley-VCH.

Although conventional single chromophore-based PS like porphyrin derivatives 228, methyl blue 229, rose bengal 230, merocyanine 540 231, and heavy metal complexes possess therapeutic capabilities for cancer, their absorption intensities and ^1^O_2_ generation efficiencies in the NIR region are not ideal. To improve NIR-mediated PDT of the existing PS, UCNPs have been developed as *in vivo* phototransducers that could emit in the visible spectrum when excited by NIR light, overlapping with the activation wavelengths of the existing PS. Liu *et al.* constructed a multilayered upconversion nanoplatform consisting of three functional layers, where the upconversion nanoparticles served as the core and light-sensitive conjugated polymers and apo-transferrin-titanocene (Tf@Tc) as shells 232. Under NIR irradiation, apparent energy transfer occurred from the core to the polymer and Tc components in the shell, producing ROS and free radicals for cancer cell killing. *In vitro* cellular assays revealed the synergistic therapeutic effect of the conjugated polymer and Tc as PS. *In vivo* studies showed that tumor growth was significantly inhibited in mice receiving the theranostic platform and NIR irradiation. Also, the tin tungstate (SnWO_4_) generated great attention, because its unique band structure has remarkable visible light active photocatalytic performance, which is not found in any other tungsten-based binary or ternary compounds. Zhang *et al.* developed hybrid nanoplatforms based on the conjugation of needle-like SnWO_4_ nanocrystals and UCNPs 233. The UCNP-emitted light of the nanoplatform was effectively absorbed by SnWO_4_, triggering the type-I PDT process to activate ROS maxima. The strong X-ray attenuation capacity of both tungsten and tin elements qualified the USWs as excellent radio-sensitizers for radiotherapy enhancement, which played a complementary role in PDT treatment. The quantum efficiency of UCNPs and consequent PDT outcomes are still suboptimal and relatively high-power intensity of the laser excitation sources are typically required. To extend NIR PS options with improved ^1^O_2_ efficiency, NIR-absorbing dyad PS based on resonance energy transfer have been developed, which have increased absorption intensity to maximize the use of absorbed photons, and greatly improve the efficiency in concomitant ^1^O_2_ generation. Huang *et al.* developed a dyad molecule based on the integration of the energy-donor moiety (distyryl-BODIPY) and PS (diiodo-distyryl-BODIPY) by click chemistry 234. The dyad molecules encapsulated with biodegradable copolymer pluronic F127-folic acid could form uniform and water-soluble nanoparticles (Figure 15A), and displayed significantly enhanced absorption and ^1^O_2_ efficiency relative to that of the acceptor moiety of the PS alone in the NIR region. *In vitro* RET-BDP-TNM showed higher fluorescence intensity than RETBDP-NNM because of the folic acid ligand at the tumor site (Figure 15B). With an exceptionally low-power NIR LED light irradiation, tumor growth (group 6) was significantly suppressed, as shown in Figure 15Cand 15D. This result indicated RET-BDP-TNM could produce strong tumor PDT.

## Conclusions and Perspective

Overall, noninvasive PDT represents an effective and highly selective tumor-ablative therapy in clinical applications. Good therapeutic effect and the possibility of the parallel application of PDT with other therapeutic protocols make it a frequently used strategy in many fields of medicine. In this review, we summarized the latest achievements and breakthroughs in constructing versatile nanoplatforms for enhanced PDT from a broad perspective. A variety of effective and proven approaches, such as stimulating a responsive release, tumor-targeting ability, O_2_ self-sufficient property, photocatalytic performance, physical effect (X-rays and surface plasmon resonance), and improvement through resonance energy transfer, were also discussed in detail.

Despite remarkable progress, there are still several considerable challenges facing the preparation of engineered nanoplatforms and their translation into clinical practice. Traditional nanoplatforms usually experience early recognition by the immune system and quick clearance from blood circulation, leading to low accumulation at the tumor site. Although some targeting nanoplatforms enhanced PDT, the local concentration was still relatively low compared to the total administered dose. Since the average distance of ^1^O_2_ diffusion is around 100 nm, targeting groups on nanoplatforms can increase their internalization in tumor cells for enhanced PDT. Thus, in the future, multiple organelle-targeting functions need to be explored for PDT, involving molecular recognition of overexpressed cellular receptors in the targeted area. Besides imparting tumor selectivity, another critical area to focus on is preventing unwanted side effects of nanoplatforms to the living organisms, thereby ensuring their compatibility and biosafety.

Currently, the performance of PS has limitations. Therefore, exploration of new PS, especially with high efficiency, is a primary focus in photodynamic research. The current complicated methods may have an impact on the application and performance of nanoplatforms. Therefore, another area of pursuit is of the relatively simple and smart methods for engineering nanoplatforms with sophisticated synthetic processes and formulations. However, another major challenge is the fundamental clinical requirements for the mass production of these nanoplatforms. Fox example, biodegraded hollow MOF-based nanoplatforms possess an efficacious PS loading capacity and maintain the high oxygen yield. Additionally, physicochemical characterization of nanoplatforms in terms of size, zeta potential, surface chemistry, colloidal stability, and, most importantly, safety protocols are needed.

The biggest payoff of versatile nanoplatforms contributes to the realization of theranostics, the integrated function of therapy and diagnostics by a single nanoformulation. For example, real-time monitoring of the therapeutic action is desired* in vitro* and even *in vivo*. It would be highly significant to provide real-time and on-site outcomes for patients for their post-treatment evaluation. Besides, it has been reported that PDT might result in cancer resistance at the cellular and molecular level. Combining PDT with different therapeutic modalities, such as chemotherapy or photothermal therapy, is crucial for further optimization of therapeutic efficiency. The mechanisms underlying the synergy between multi-modal theranostics need to be explored. In particular, the therapeutic effect of 1+1 = >2 must be exerted rather than the additive effect of the individual features, the mechanism of synergy between multi-modal theranostics also needs to be deeply explored. The combination of different modalities is expected to provide functional compensation, diagnostic accuracy, and improved therapeutic efficiency.

Finally, most of the tumors are located deep inside the body rather than on the surface, requiring improved deep penetrating ability of PDT for deep-seated therapy. Immunotherapy has recently gained immense credence for eliminating distant metastatic cancer cells and remove residual cancer cells in the body. Thus, the synergistic use of deep tissue PDT and immunotherapy offers unique advantages in treating both deep-seated primary tumors and distant metastatic tumors. The successful translation of these nanoplatforms to clinical applications is anticipated in the near future upon satisfactory tackling of the current critical issues.

## Figures and Tables

**Table 1:** Targeting and responsive nanoplatforms for enhanced PDT

Nanoplatforms design	PS	Enhanced mechanism	Improved therapeutics	Ref
Chimeric peptide	Ce6	Cell membrane target	PDT	53
Peptide	PpIX	pH-driven membrane-anchoring	PDT	54
SiO_2_-TAT/RGD	Ce6	Nuclear-targeted delivery	PDT	56
Coordination polymers	Ce6	Nuclear-targeted drug delivery	PDT	57
Supramolecular nanocarriers	Ce6	Mitochondria-target	PDT	62
Chimeric peptide	PpIX	Mitochondria/plasma membrane-target	PDT	63
Lyso-Ru-NO@FA@C-TiO_2_	C-TiO_2_	Lysosome-targeted delivery	PDT	66
Iridium(III) complexes	Ir-P(ph)_3_/Ir-alkyl	Mitochondria /lysosome target	PDT	67
C-Phycocyanin-ZnPc conjugates	ZnPc	Macrophage-target	PDT	69
Pseudopolyrotaxane micelles	PpIX	pH-responsive release	PDT	88
Polymeric micelles	Ce6	pH-responsive/EGFR targeting peptides	PDT	89
Coordination polymer nanoparticles	ZnPc	Charge-reversal	PDT	92
Supramolecular amphiphiles	Ce6	Redox-responsive	PDT	101
Amphiphilic polymers	Ce6	Redox stimulation	PDT	102
Nano-MOF with Cu^II^	Porphyrin	Reducing of GSH	PDT	105
BSA-MBPB	Methylene blue	H_2_O_2_-activatablity	PDT	106
HA-Ce6 conjugation	Ce6	Hyaluronidase	PDT	109
Polymer vesicles	Nile blue	Enzyme simulation	PDT	111
Amphiphilic block copolymer	Ce6	Singlet oxygen-sensitive	Chemo-PDT	117
Polymeric micelles	Ce6	Hypoxia/Singlet oxygen responsive	PDT	118
Dopamine-reduced graphene oxide	Ce6	Photothermal responsive	PDT/PTT	133

**Table 2:** Chemical effects of nanoplatforms for enhanced PDT

Nanoplatforms design	PS	Enhanced mechanism	Improved therapeutics	Ref
Perfluorotributylamine-HSA	IR780	Perfluorotributylamine affinity O_2_	PDT	146
Perfluorohexane @Ce6@O_2_ nanodroplets	Ce6	Perfluorohexane affinity O_2_	PDT	147
Covalent organic polymers with PFC	Porphyrin	Perfluoro-15-crown- 5-ether affinity O_2_	PDT	148
Fluorocarbon-functionalized nanoparticles	IR780	pH-sensitive fluorocarbon/ iRGD	PDT	149
Lipid-polymer/PFC	Ce6	Perfluoro-octan-1-ol affinity O_2_	PDT	150
Upconversion nanoparticles with RBC	Rose bengal	RBC deliver O_2_	PDT	151
RBC/ammonium bicarbonate/DOX	ICG	RBC deliver O_2_	Chem/ PTT /PDT	152
Amphiphilic triblock copolymers	ZnPc	Hemoglobin deliver O_2_	PDT	153
Hemoglobin conjugated polymer nanoparticles	Luminol	Hemoglobin deliver O_2_	PDT	154
Catalase-loaded hierarchical zeolite	Methylene blue	Catalase generate O_2_	PDT	155
HSA-Ce6-catalase-PTX nanoparticles	Ce6	Catalase generate O_2_	Chemo-PDT	157
PTX/ICG-nanovehicles@Au@ catalase nanoparticles	ICG	Catalase generate O_2_	PDT/PTT	159
UCNPs @ZIF-8@catalase	Methylene blue	Catalase generate O_2_	PDT	160
Catalase@S/Ce6-CTPP/DPEG	Ce6	Catalase generate O_2_	Immuno-PDT	161
Catalase-entrapped nanocapsules	Porphyrin	Catalase generate O_2_	PDT	162
Catalase-integrated hyaluronic acid	Ce6	Catalase generate O_2_	PDT	163
Black phosphorus/MnO_2_	Black phosphorus	MnO_2_ generate O_2_	PDT	164
Magnetofluorescent Carbon Dot	Phthalocyanine	MnO_2_ nanodots generate O_2_	PDT	167
Mn-Cdots/DOX-loaded mesoporous silica-coated gold cube nanocomposites	Phthalocyanine	MnO_2_ nanodots generate O_2_	PDT/PTT	168
PLGA/hematoporphyrin monomethyl ether @MnO_2_	Porphyrin	MnO_2_ generate O_2_	PDT	169
Upconversion composite nanoparticles	Ce6	MnO_2_ generate O_2_	PDT	170
Ce6@MnO_2_-PEG nanoparticles	Ce6	MnO_2_ generate O_2_	PDT	167
Hydrangea MnO_2_/DOX	aza-BODIPY	MnO_2_ generate O_2_	PDT	175
Semiconducting hybrid nanoparticles	PCPDTBT	MnO_2_ generate O_2_	PDT	176
MnFe_2_O_4_-anchored mesoporous silica	Ce6	Fenton reaction	PDT	178
MnFe_2_O_4_@Metal-organic frameworks	Porphyrin	Fenton reaction/ consume GSH	PDT	179
MoS_2_/rGO-MnO_2_-PEG	Reduced graphene oxide	p-n heterojunction/ MnO_2_ generate O_2_	PDT	180
F127-MnO_2_-ZIF@DOX/C_3_N_4_	C_3_N_4_	MnO_2_ nanodots generate O_2_	Chemo-PDT	181
Pt Metal-organic frameworks	Porphyrin	Pt nanozymes produce O_2_	PDT	185
Pd@Pt-PEG-Ce6	Ce6	Pt nanozymes produce O_2_	PDT/PTT	186
H_2_O_2_/PLGA	IR780	H_2_O_2_ release O_2_	PDT	187
UCNP@mSiO_2_@ZIF-90-DOX-PEGFA	Rose bengal	ZIF-90 reserve O_2_	Chemo-PDT	188
UCNP with hyperbaric oxygen	Rose bengal	Hyperbaric oxygen supply O_2_	PDT	189
Carbon dots/TiO_2_ nanotubes	Carbon dots	Carbon dots enhance light absorption	PDT	195
TiO_2_-ruthenium nano-photosensitizer	Organometallic ruthenium	N_3_ injected electrons into TiO_2_	PDT	196
Carbon-dot-doped C_3_N_4_/ amphipathic polymer	PpIX	C_3_N_4_ split water to generate O_2_	PDT	199
Fe^III^-doped-C_3_N_4_ nanofusiform	Methylene blue	H_2_O_2_-activatable, O_2_-evolving, mitochondrial- target	PDT	200
Cu^II^-doped-C_3_N_4_	C_3_N_4_	Reduce GSH	PDT	201
Ultrathin Graphdiyne oxide/iRGD peptide-modified RBC	Graphdiyne oxide	sufficient O_2_/ tumor cell targeting /penetrating	PDT/PTT	202

**Table 3:** Physical effects of nanoplatforms for enhanced PDT

Nanoplatforms design	PS	Enhanced mechanism	Improved therapeutics	Ref
Ion-incorporated silicate nanoscintillators	Rose bengal	X-rays transmit the energy to PS	PDT	213
Mesoporous LaF_3_:Tb nanoparticles	Rose bengal	X-rays transmit the energy to PS	PDT	216
LiLuF_4_:Ce@SiO_2_@Ag_3_PO_4_@Pt(IV)	LiLuF_4_:Ce	X-rays transmit the energy to PS	PDT	218
CeF_3_:Gd^3+^,Tb^3+^@SiO_2_	Rose bengal	X-rays transmit the energy to PS	PDT/ radiotherapy	219
Gold nanorods@ SiO_2_	(Eu) complexes	X-rays transmit the energy to PS	PDT/PTT	220
PLGA /Gold nanoparticles	Verteporfin	X-rays transmit the energy to PS	PDT	222
Silica-gold nanorod	ICG	SPR	PDT	225
UCNP-gold nanorod	Methylene blue	SPR	PDT	226
Gold nanoparticles with black phosphorus	Black phosphorus	SPR	PDT/PTT	227
Multilayered upconversion	PFSBT	Resonance energy transfer	PDT	232
SnWO_4_-based nanohybrids	SnWO_4_	Resonance energy transfer	PDT/ radiotherapy	233
Dyad molecule with pluronic F-127-folic acid	BODIPY	Resonance energy transfer	PDT	234
